# Supplementary material for: Separation of Anionic Chlorinated Dyes from Polluted Aqueous Streams Using Ionic Liquids and Their Subsequent Recycling
Source: Int J Mol Sci. 2023 Jul 31;24(15):12235. doi: 10.3390/ijms241512235 (PMC10418802; doi:10.3390/ijms241512235)
Supplement: Supplementary file 1 [file ijms-24-12235-s001.zip › ijms-2498251-supplementary.pdf]

Supplementary Materials

Separation of Anionic Chlorinated Dyes from Polluted Aqueous Streams Using Ionic Liquids and Their Subsequent Recycling

Barbora Kamenická [1], Petr Švec [2], and Tomáš Weidlich [1*]

[1] Chemical Technology Group, Institute of Environmental and Chemical Engineering, Faculty of Chemical Technology, University of Pardubice, Studentská 573, 532 10 Pardubice, Czech Republic.

[2] Department of General and Inorganic Chemistry, Faculty of Chemical Technology, University of Pardubice, Studentská 573, 532 10 Pardubice, Czech Republic.

* Correspondence: tomas.weidlich@upce.cz; Tel.: +420-46-603-8049

Academic Editor: name

Received: ….. 2023; Accepted: … 2023; Published: …..2023

Number of pages: 29

Number of Tables: 2

Number of Schemes: 2

Number of Figures: 14

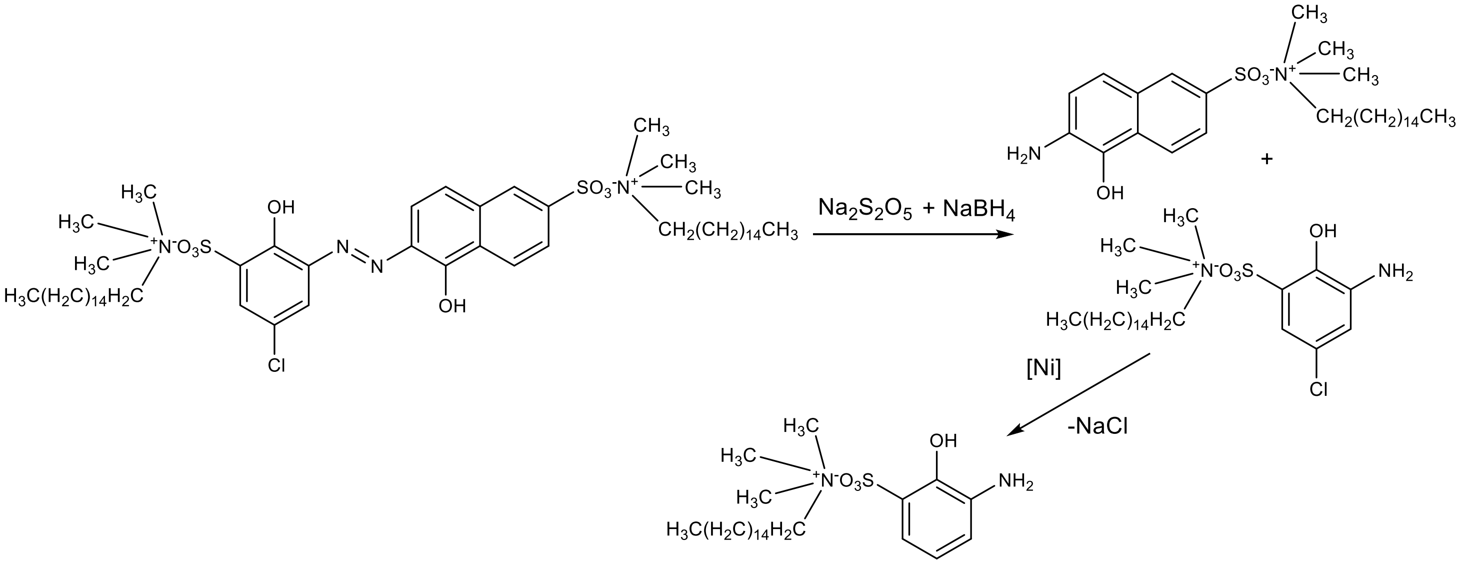

Scheme S1. Proposed reaction pathway of the reductive degradation and subsequent HDC of ion pair $(AlkMe_3N)_2$.MB9 using $NaBH_4/Na_2S_2O_5$ in combination with the Al-Ni alloy.

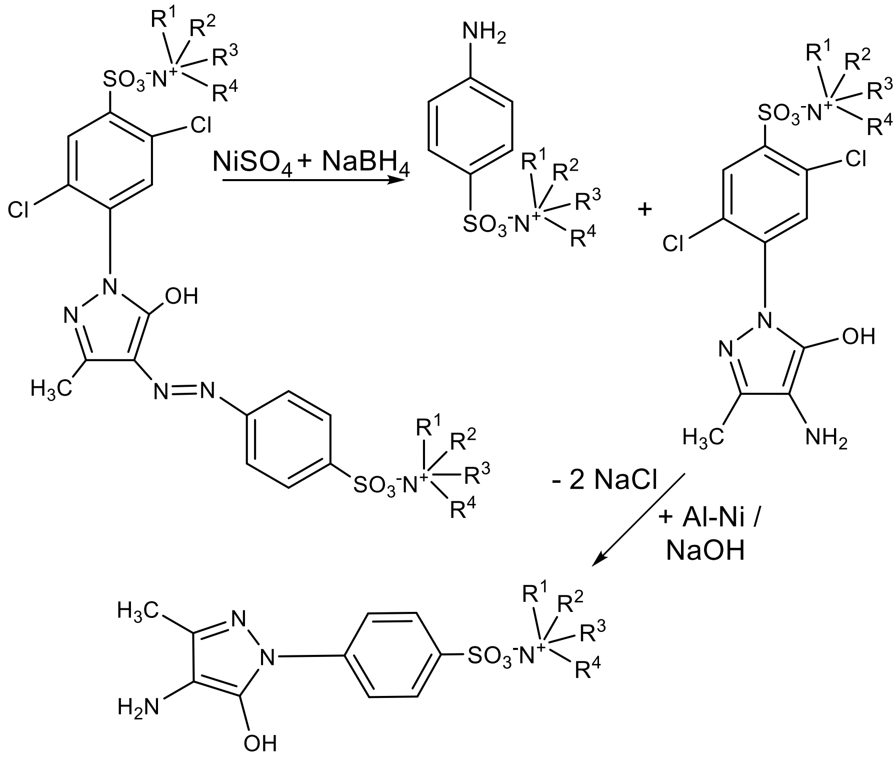

R = $-CH_3$, $-CH_2(CH_2)_nCH_3$, $-CH_2Ph$

Scheme S2. Proposed reaction pathway of the reductive degradation and subsequent HDC of ion pair $(R_4N)_2$.AY17 using $NaBH_4/NiSO_4$ in combination with the Al-Ni alloy.

Table S1. A comparison of acid dyes extraction and ion exchange methods using ILs.

Method	Type of dye	Concentration of dye (mg/L)	ILs [a]	Dosage of ILs (g/L)	Removal efficiency (%)	Ref.
Extraction	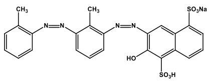 Acid Red Dye *	5	$[p_{14}][tf_2N]$	700	98	[16]
Extraction	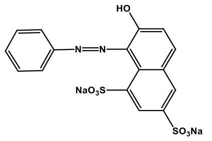 Acid Orange 52	1000	$[C_8mim][PF_6]$	246.9	99	[17]
Extraction	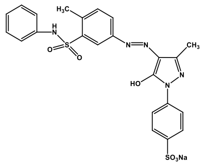 Acid Yellow RN	100	$[Bmim][PF_6]$	1380	92.87	[18]
Extraction	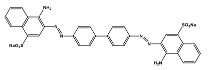 Congo Red	250	$[Hmim][tf_2N]$	40	95	[19]
Extraction	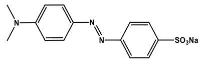 Methyl Orange	40	[Capryl3MeN][SCN]	276	89.09	[20]
Extraction	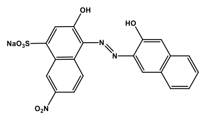 Eriochrome Black T	250	$[C_6mim][BF_4]$	43.1	99.65	[21]
Extraction	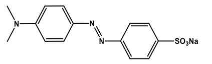 Methyl Orange	1000	$[PC_6C_6C_6C_{14}]$ $[N(CN)_2]$	20	100	[22]
Extraction	„Royal acid dye´´ (from Brasilian textile industry) wastewater)	50	$[PC_6C_6C_6C_{14}]$ $[C_9H_{19}COO]$	0.125	94	[23]
Ion exchange	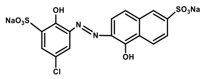 Mordant Blue 9	2111	Luv. Mono LS	17.8	90	This work

Method	Type of dye	Concentration of dye (mg/L)	ILs [a]	Dosage of ILs (g/L)	Removal efficiency (%)	Ref.
Ion exchange	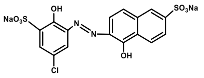 Mordant Blue 9	2111	BzkoniumCl	17.3	82.1	This work
Ion exchange	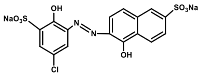 Mordant Blue 9	2111	A336	20.2	93.2	This work

[a] *Abbreviations:* [p_{14}][tf_2N] – N-butyl,N-methyl pyrrolidiniumbis(trifluoromethanesulfonyl) imide; [C_8mim][PF_6] – 1-octyl-3-methylimidazolium hexafluorophosphate; [Bmim][PF_6] – 1-Butyl-3-methylimidazolium hexafluorophosphate; [Hmim][tf_2N] – 1-Hexyl-3-methylimidazoliumbis(trifluoromethylsulfonyl) imide; [Capryl3MeN][SCN] – Tricaprylmethylammonium thiocyanate; [C_6mim][BF_4] – 3-methylimidazolium tetrafluoroborate; [$PC_6C_6C_6C_{14}$][$N(CN)_2$] – Trihexyl(tetradecyl)phosphonium bis(trifluoromethanesulfonyl) dicyanamide; [$PC_6C_6C_6C_{14}$][$C_9H_{19}COO$] – Trihexyl(tetradecyl)phosphonium 4-methylnonanoate.
Notes: *Commercial name not specified

Table S2. Comparison of costs of various reductive methods of ion pairs (calculated per mol of MB9).

Applied reductive agent	Reactants / 1 mol of $(R_4N)_2$.MB9	Price of method per mol of $(R_4N)_2$. MB9 *
$NaBH_4$	100 mol $NaBH_4$	522 $
Al-Ni alloy/NaOH	64 mol Al in Al-Ni alloy / 300 mol NaOH	1160 $
$NaBH_4$/$NiSO_4$	50 mol $NaBH_4$ (12% $NaBH_4$ in 14M NaOH) / 10 mol $NiSO_4$	327 $
$NaBH_4$/$NiSO_4$/Al-Ni alloy	50 mol $NaBH_4$ (12% $NaBH_4$ in 14M NaOH) / 10 mol $NiSO_4$ + 20 mol Al in Al-Ni alloy	639 $

* Costs are calculated with respect to the lowest available price according to global market suppliers, see [9].

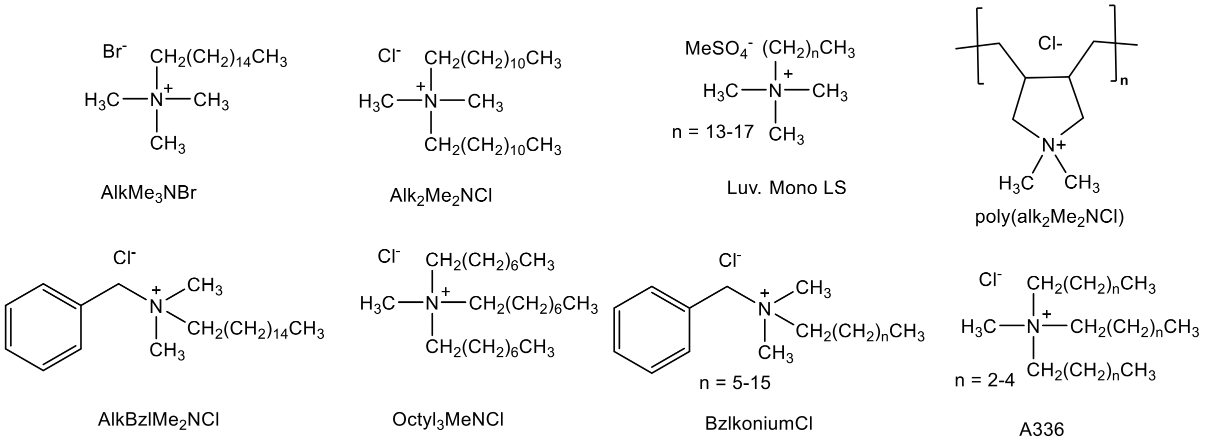

Figure S1. Structural drawings of tested R_4NX.

($AlkMe_3NBr$ – cetyltrimethylammonium bromide; Alk_2Me_2NCl – dilauryldimethylammonium chloride; $AlkBzlMe_2NCl$ – benzyldimethylhexadecylammonium chloride; $Octyl_3MeNCl$ – trioctylmethylammonium chloride; Luv. Mono LS – Luviquat Mono LS; BzlkoniumCl – Benzalkonium chloride; poly(Alk_2Me_2NCl) – poly(dialkyldimethylammonium chloride); A336 – Aliquat 336)

Figure S2. ^{1}H NMR spectra of evaporated residue of CH_2Cl_2 extracts of MB9 (DMSO-d_6).

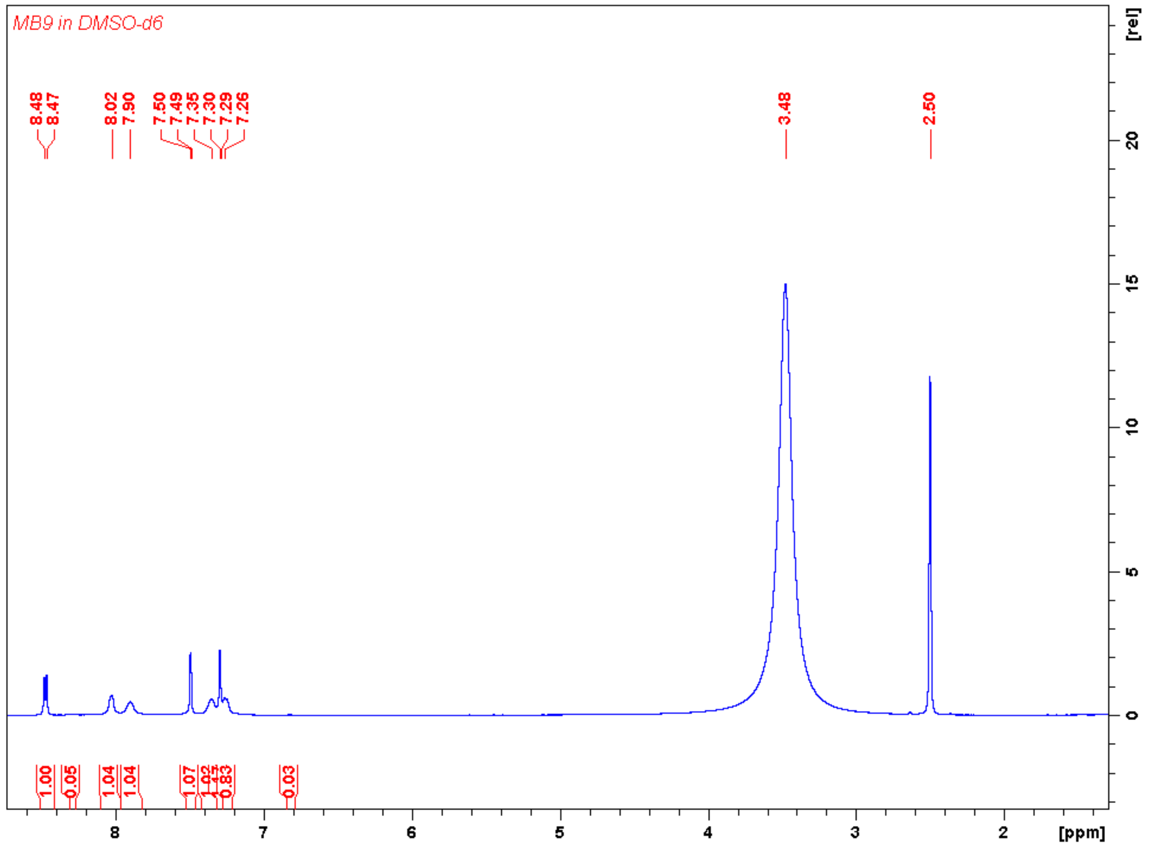

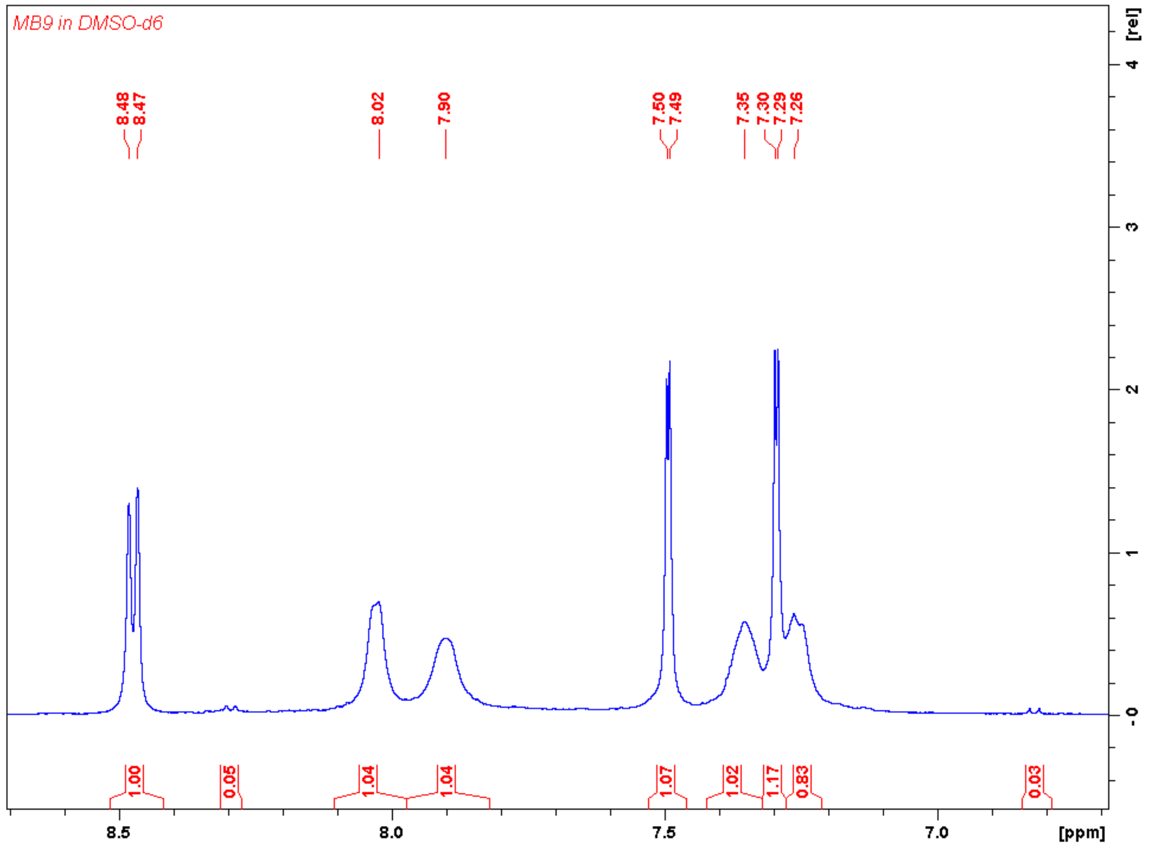

Figure S3. ^1H NMR spectra of evaporated residue of CH_2Cl_2 extracts obtained after the reaction of MB9 in aqueous solution with $AlkMe_3NBr$ (DMSO-d6).

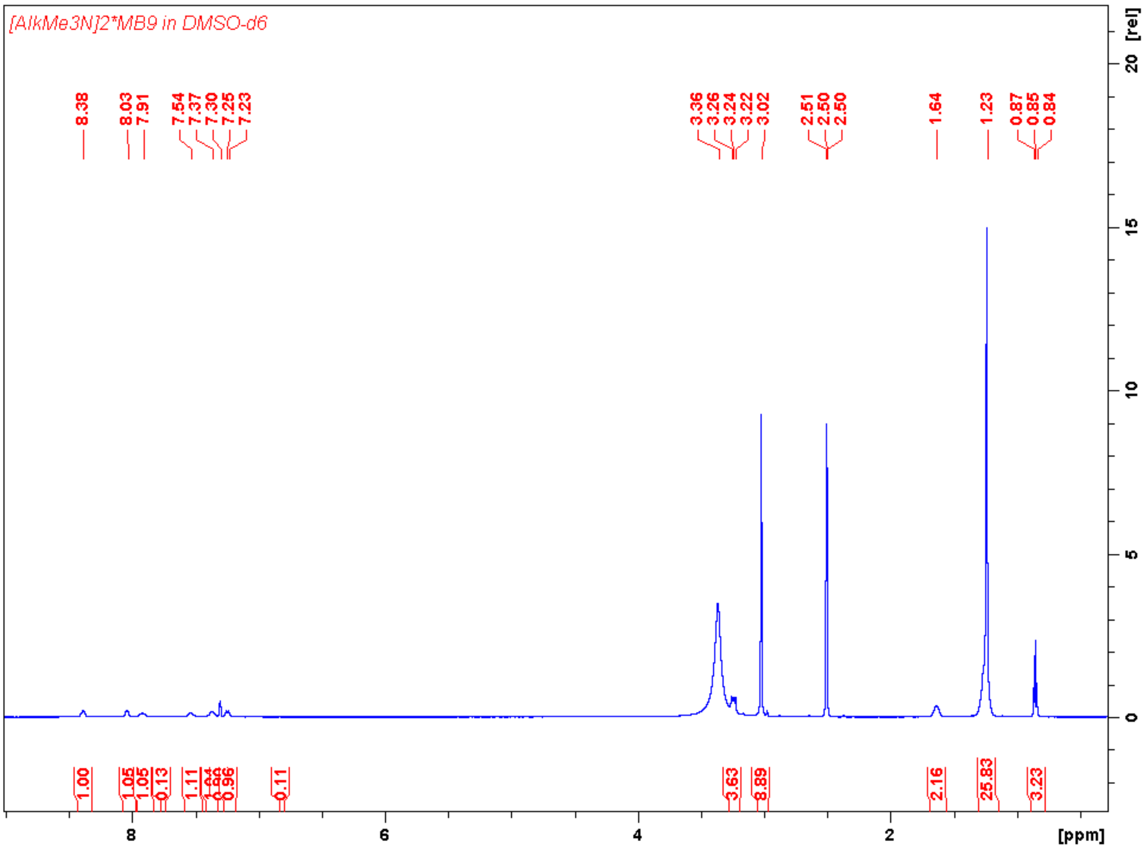

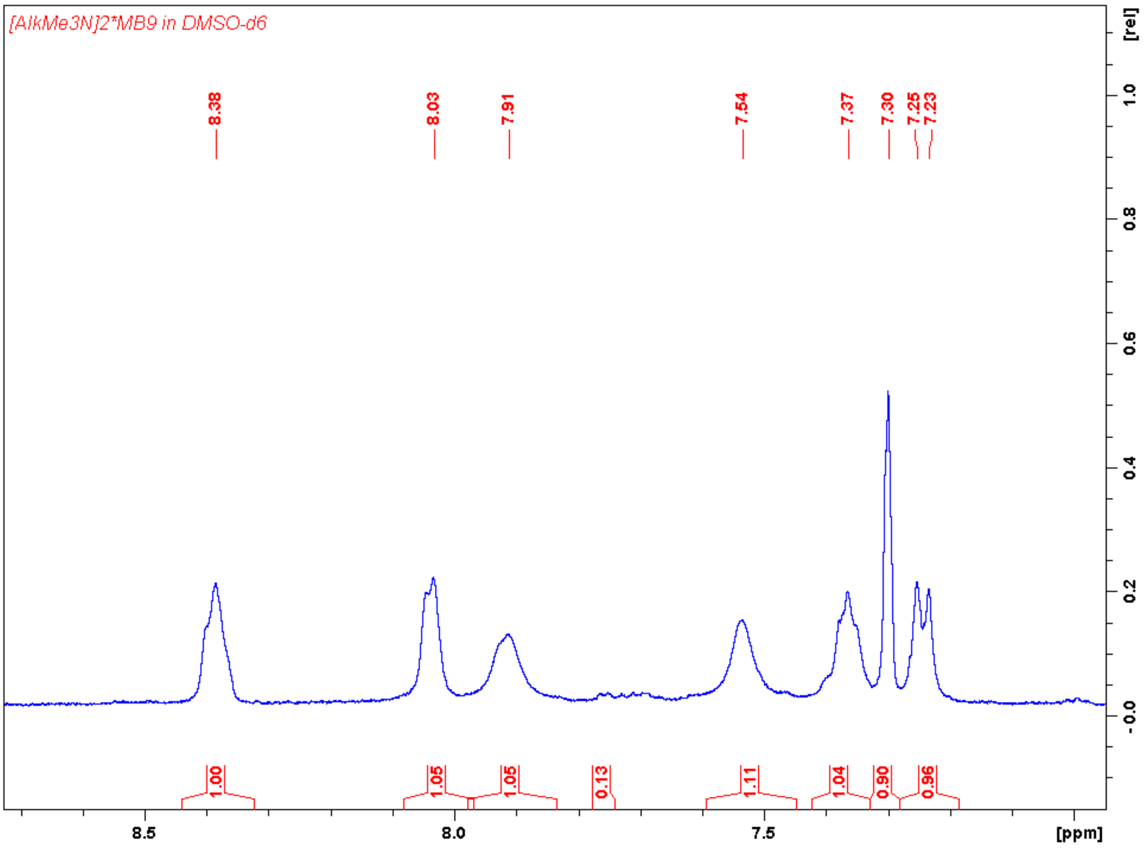

Figure S4. ^{1}H NMR spectra of evaporated residue of CH_2Cl_2 extracts obtained after the reaction of MB9 in aqueous solution with $AlkBzMe_2NCl$ ($CDCl_3$).

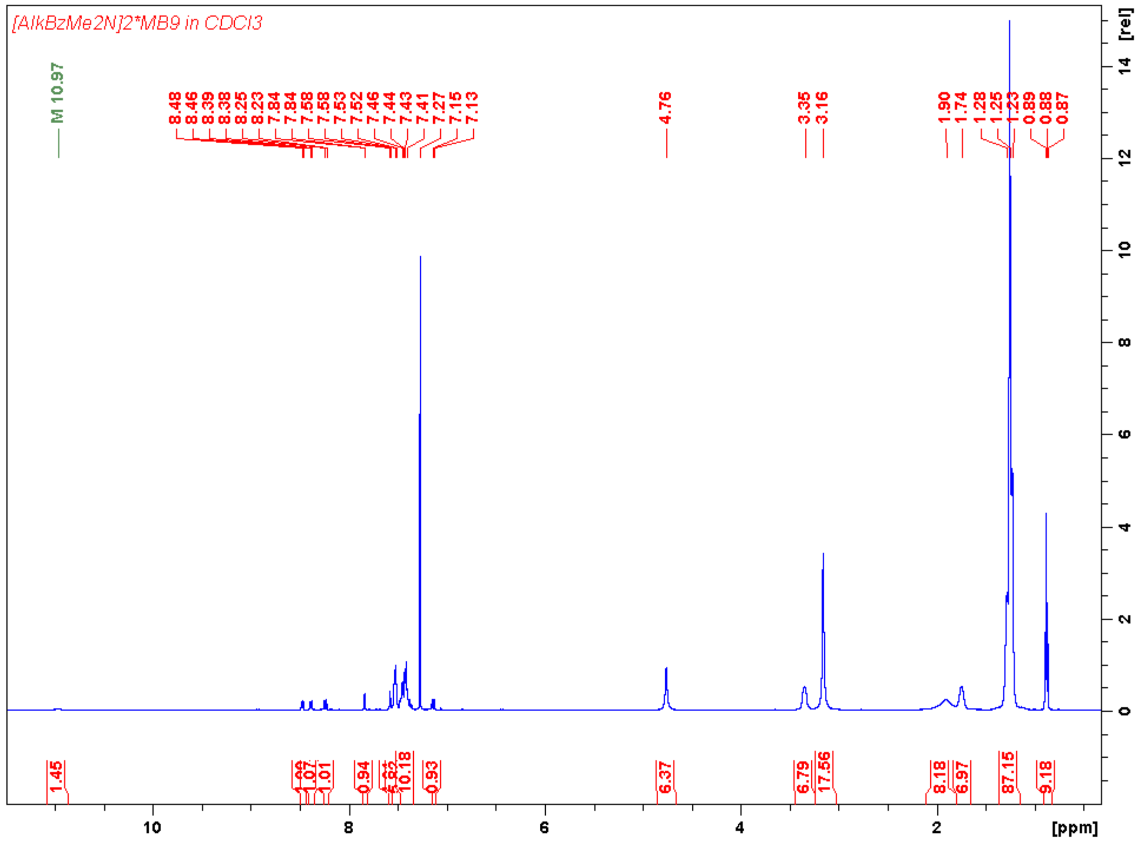

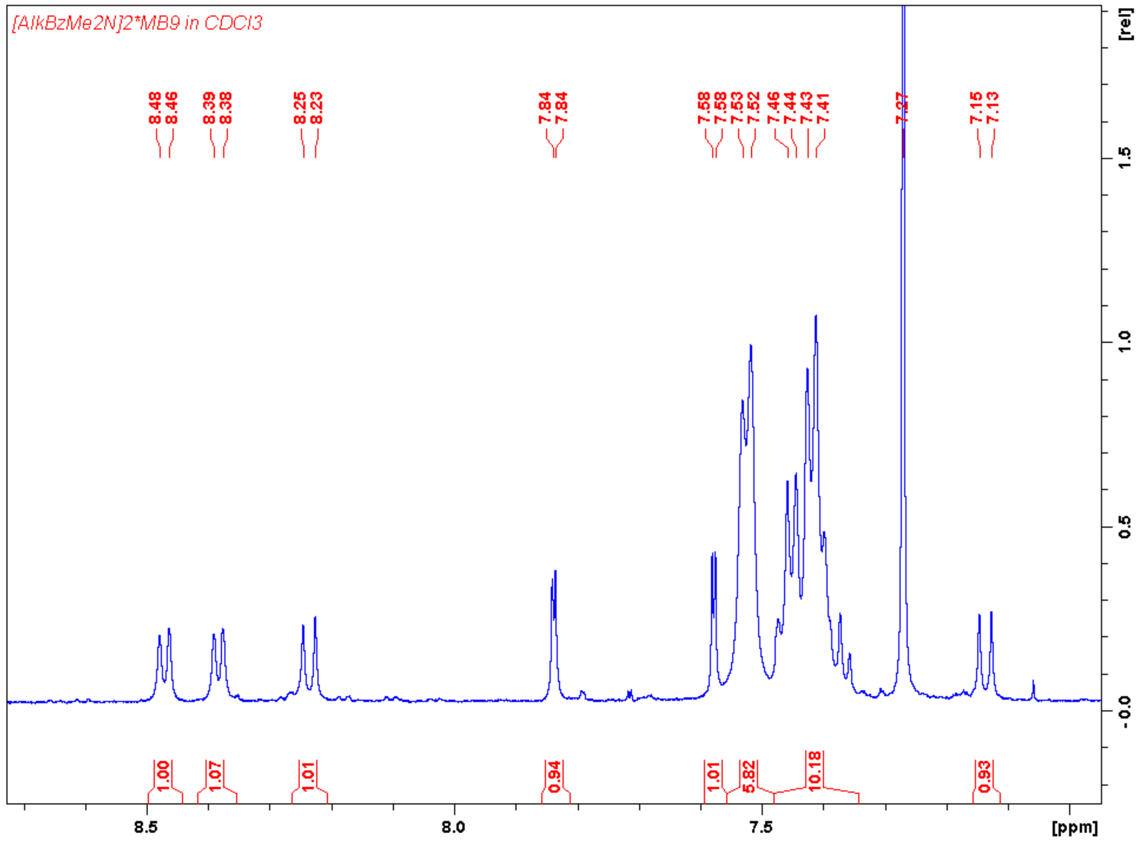

Figure S5. ^{1}H NMR spectra of evaporated residue of CH_2Cl_2 extracts obtained after the reaction of MB9 in aqueous solution with $Octyl_3MeNCl$ ($CDCl_3$).

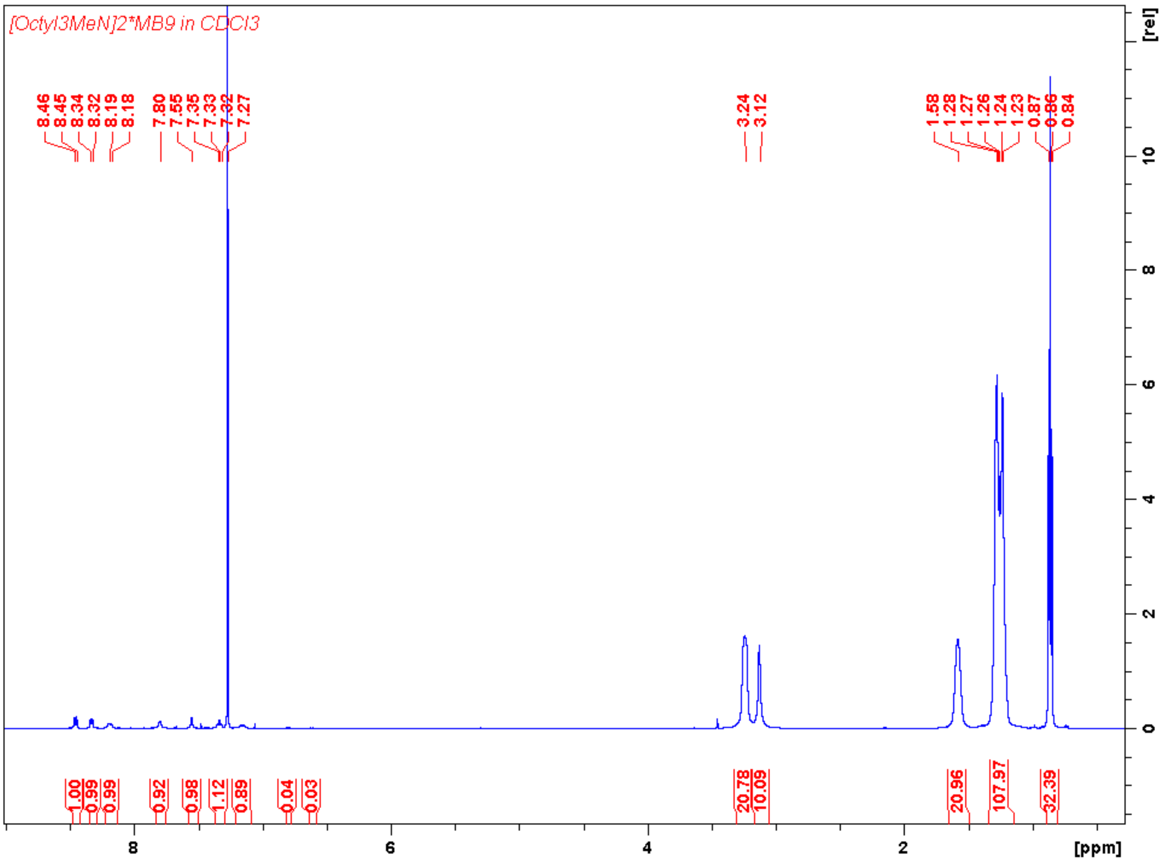

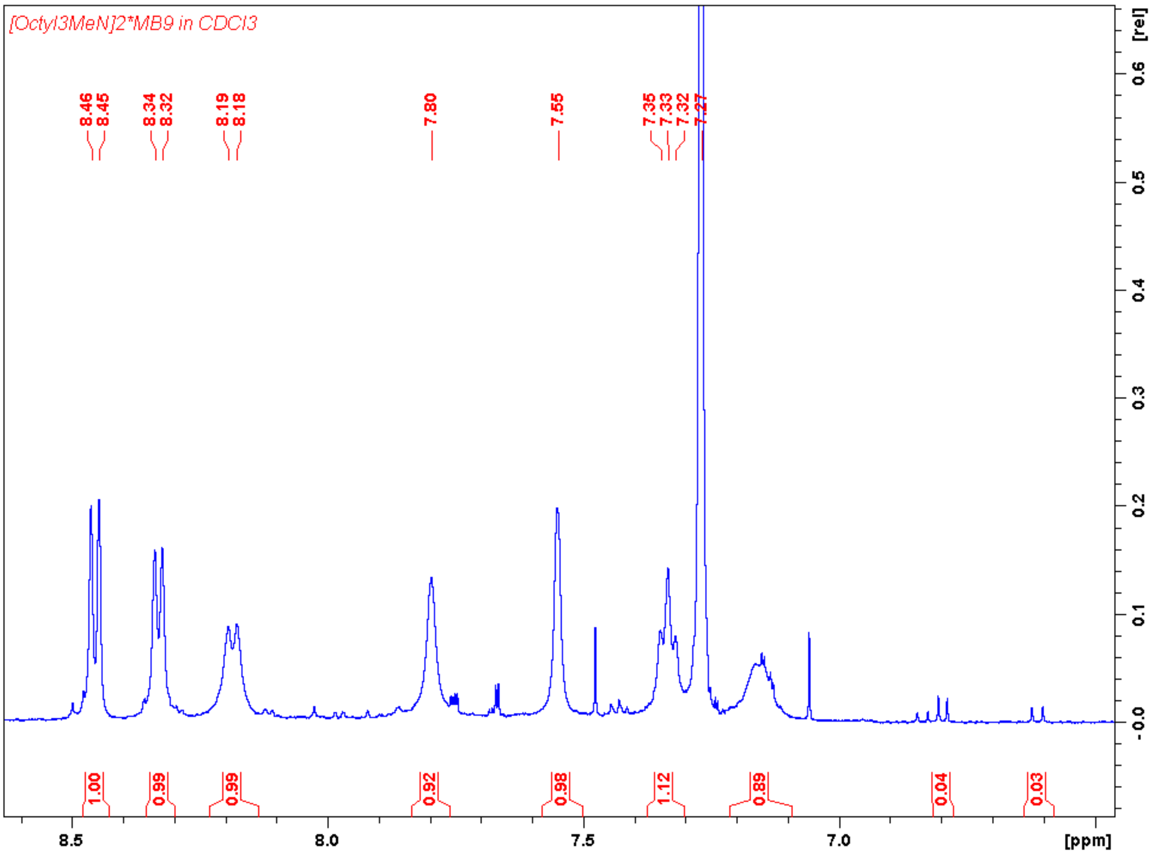

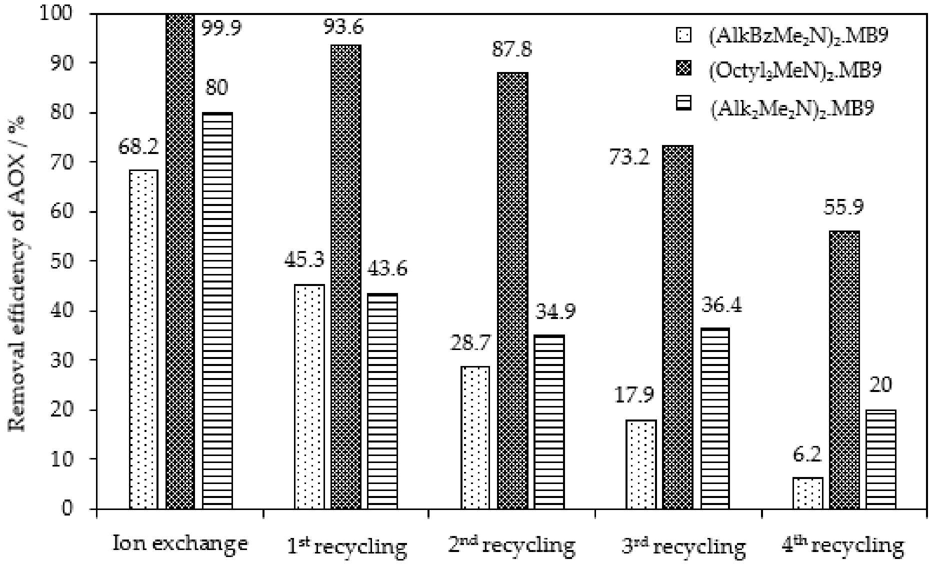

Figure S6. Results of the effectiveness of the AOX removal of $(R_4N)_2$.MB9 within four recycling steps. (Ion pair formation: 1.2 mmol of R_4NX in 50 mL CH_3OH + 0.5 mmol of MB9 in 120 mL H_2O / Reduction: addition of 25 mmol $NaBH_4$ to ion pair solution in the 1st-2nd cycle and 50 mmol of $NaBH_4$ in the 3rd-4th cycle / Recycling: generated ion pair species in 50 mL CH_3OH + 0.5 mmol of MB9 in 120 mL of H_2O; reaction time of the ion exchange 1 hour and reaction time of the reduction 2 hours).

Figure S7. a) LC chromatogram of the LC-MS analysis of the reaction mixture obtained after the reductive degradation of ion pair $(AlkMe_3N)_2$.MB9 using $NaBH_4/Na_2S_2O_5$ and $NiSO_4$.

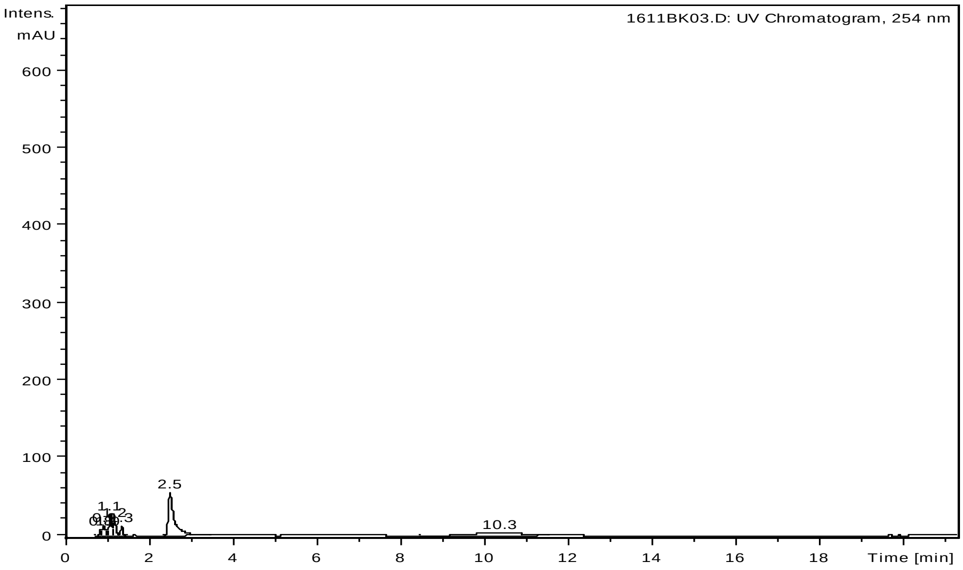

b) Qualitative LC-MS analysis of the reaction mixture obtained after the reductive degradation of ion pair $(AlkMe_3N)_2$.MB9 using $NaBH_4/Na_2S_2O_5$ and Al-Ni/NaOH.

RT (min)	*MW*	*Structure*
2.5	223	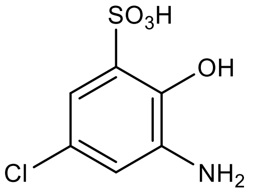
10.3	430	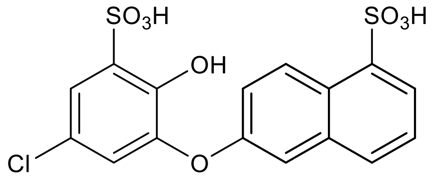

Note: Structures of the detected isomer compounds could not be to confirmed due to the lack of corresponding standard compounds.

c) Mass spectra (EI, 70 eV) of analyzed components.

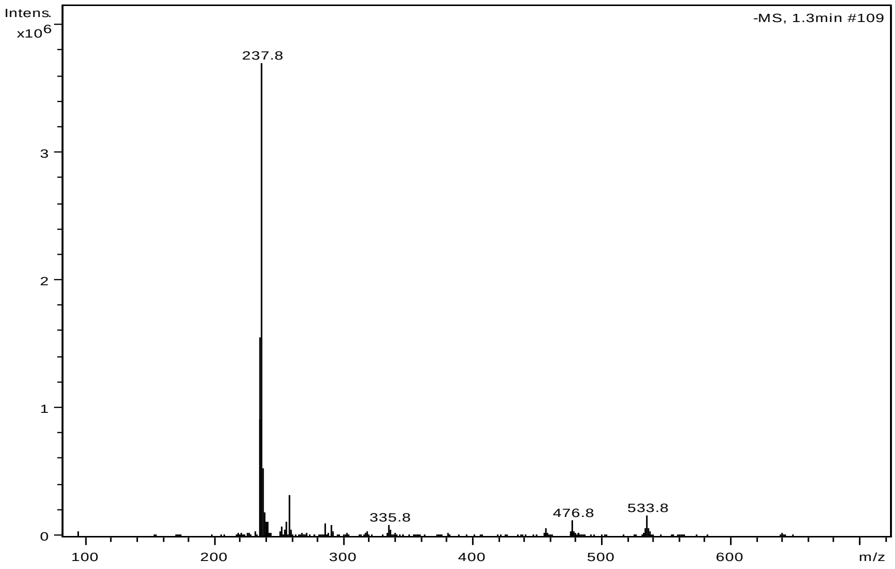

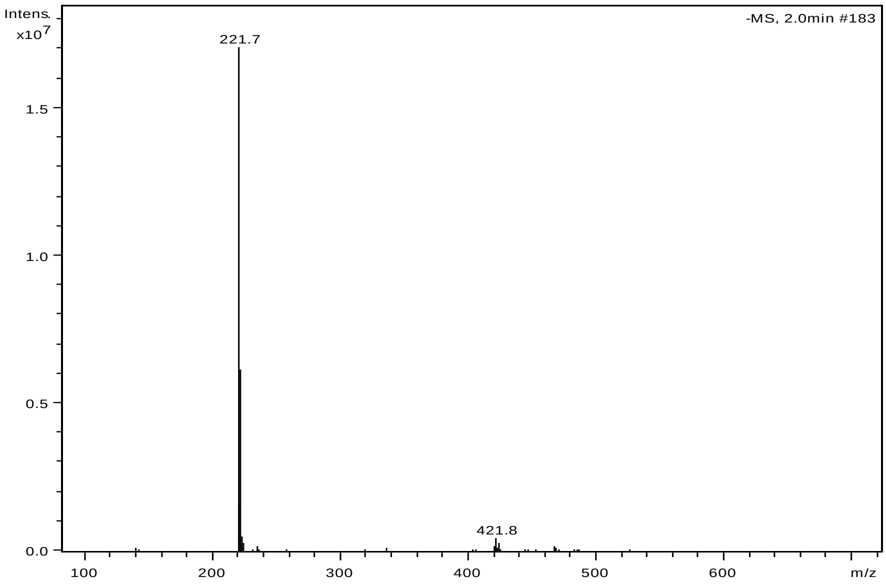

Figure S8. a) LC chromatogram of the LC-MS analysis of the reaction mixture obtained after the reductive degradation of ion pair $(AlkMe_3N)_2$.MB9 using $NaBH_4/Na_2S_2O_5/NiSO_4$ and Al-Ni/NaOH.

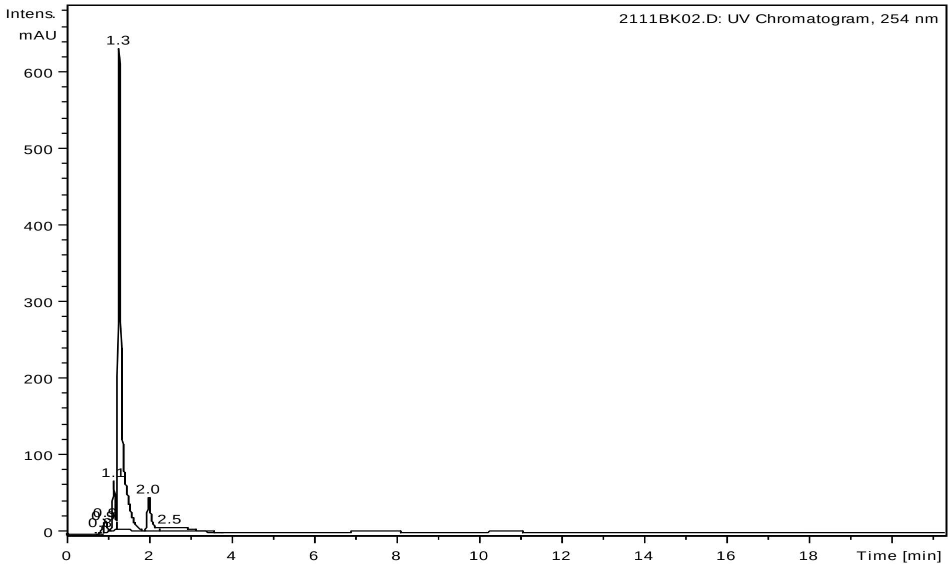

b) Qualitative LC-MS analysis of the reaction mixture obtained after the reductive degradation of ion pair $(AlkMe_3N)_2$.MB9 using $NaBH_4/Na_2S_2O_5$ and Al-Ni/NaOH.

RT (min)	***MW***	***Structure***
1.3	239	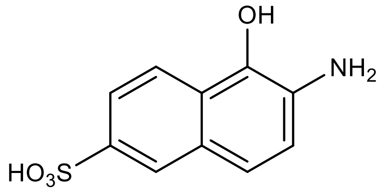
2.0	223	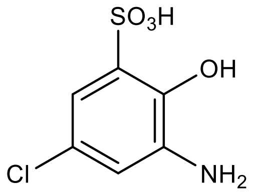
2.5	223	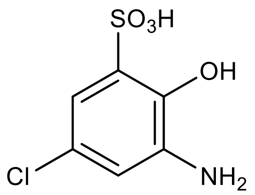

Note: Structures of the detected isomer compounds could not be to confirmed due to the lack of corresponding standard compounds.

c) Mass spectra (EI, 70 eV) of analyzed components.

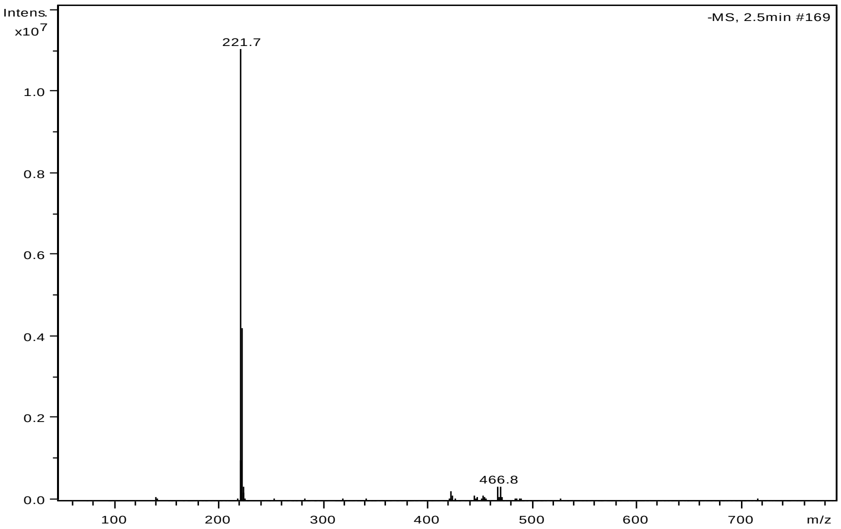

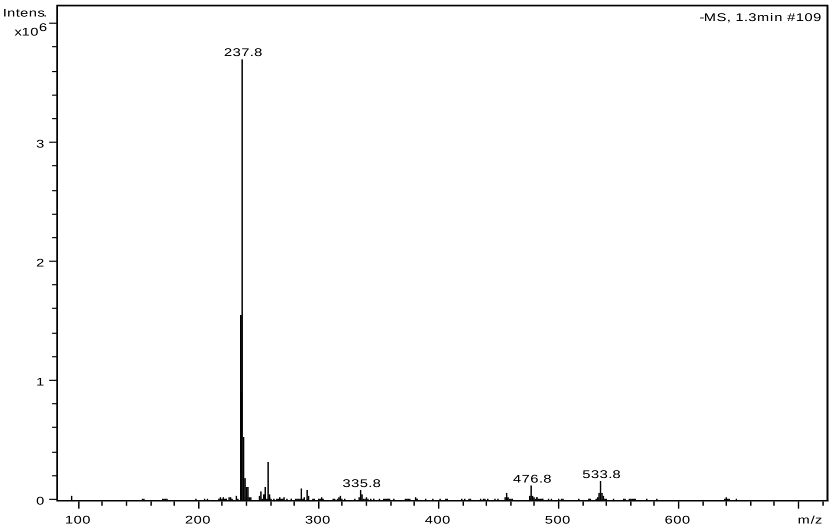

Figure S9. a) LC chromatogram of the LC-MS analysis of the reaction mixture obtained after the reductive degradation of ion pair (Luv. Mono LS)2.MB9 using $NaBH_4/NiSO_4$.

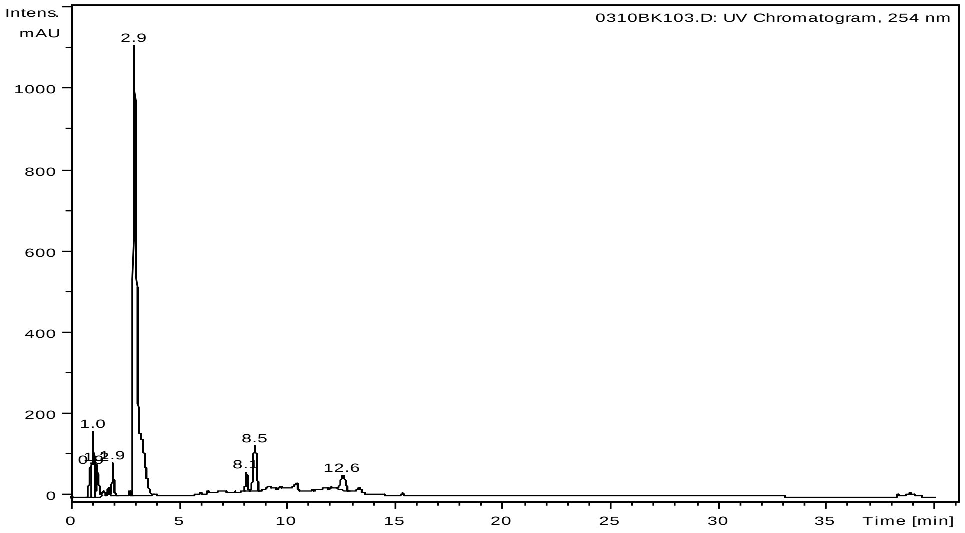

b) Qualitative LC-MS analysis of the reaction mixture obtained after the reductive degradation of ion pair (Luv. Mono LS)2.MB9 using $NaBH_4/NiSO_4$.

RT (min)	***MW***	***Structure***
0.9	-	detected chlorinated structure
1.0	-	detected chlorinated structure
1.2	-	detected chlorinated structure
1.9	-	detected chlorinated structure
2.9	223	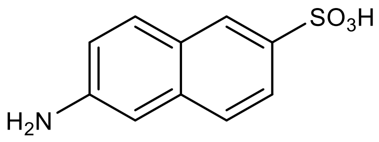
8.1	-	detected chlorinated structure
8.5	-	detected chlorinated structure
12.6	424	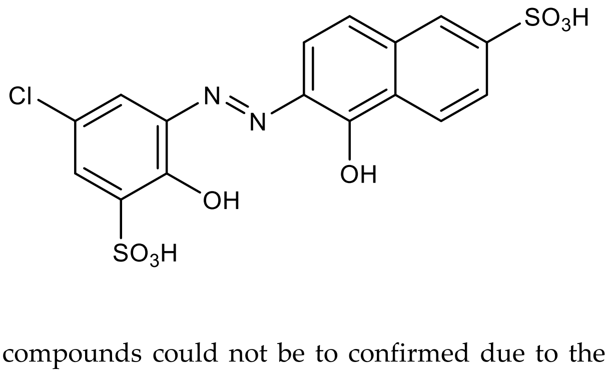

Note: Structures of the detected isomer compounds could not be to confirmed due to the lack of corresponding standard compounds.

c) Mass spectra (EI, 70 eV) of analyzed components.

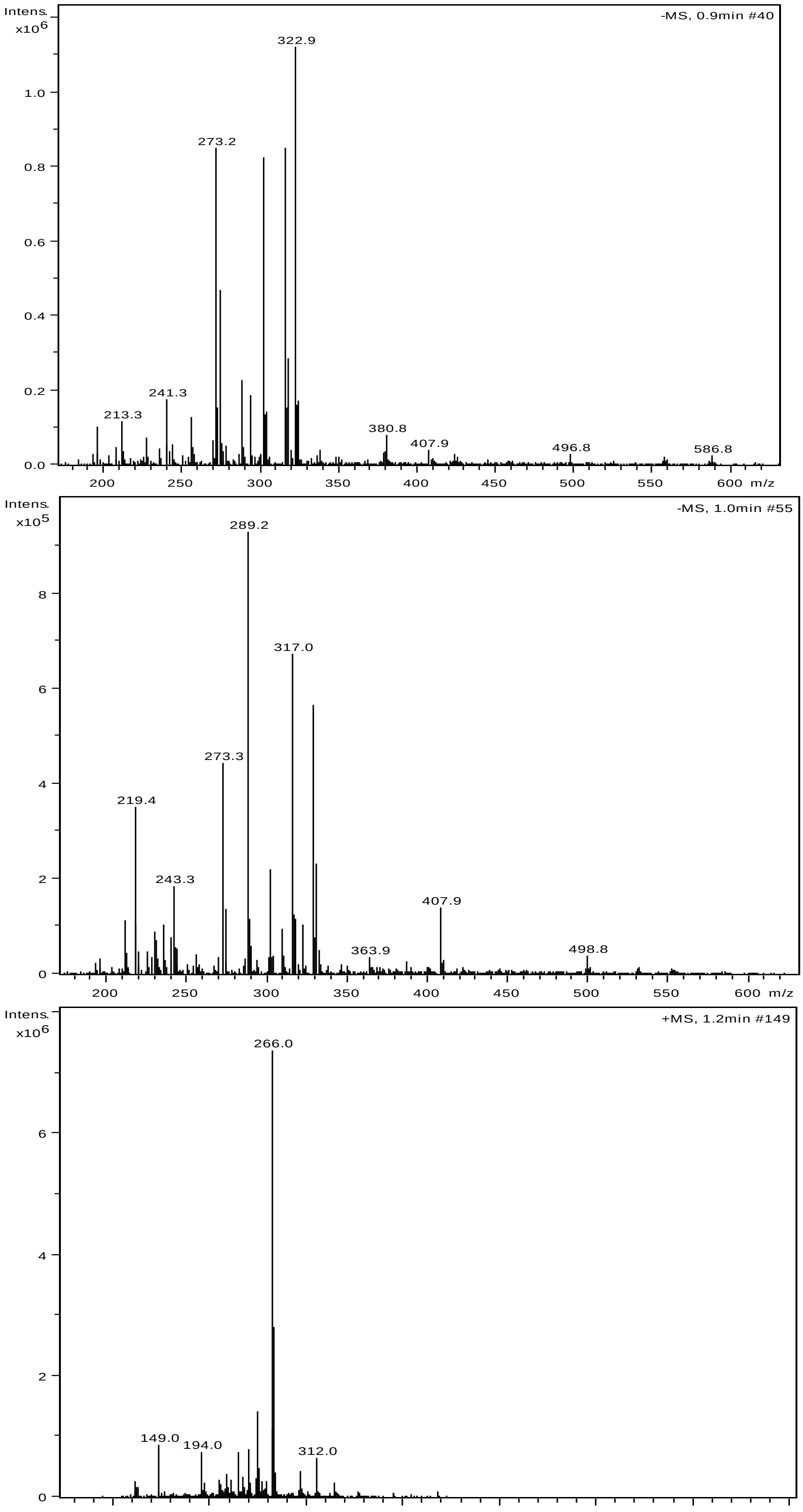

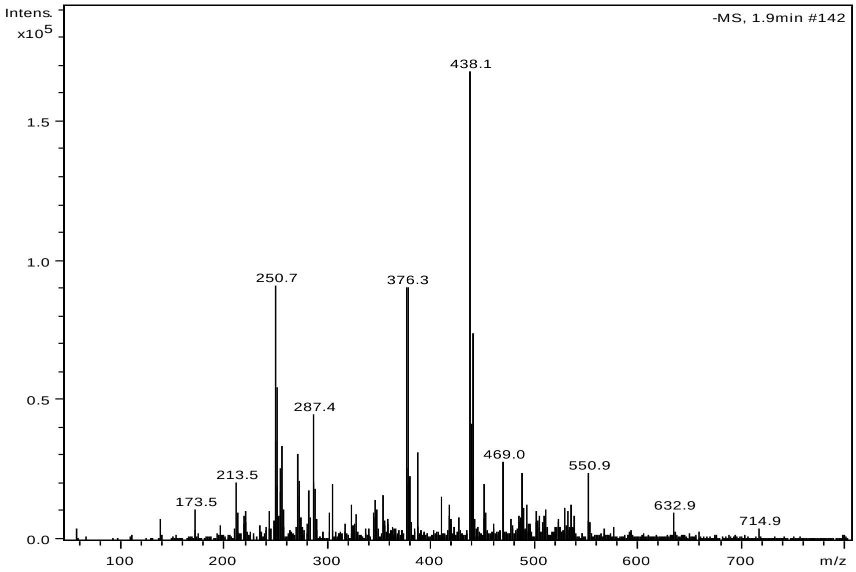
Intens.
x10^5
-MS, 1.9min #142
438.1
250.7
376.3
287.4
469.0
550.9
213.5
173.5
632.9
714.9
m/z

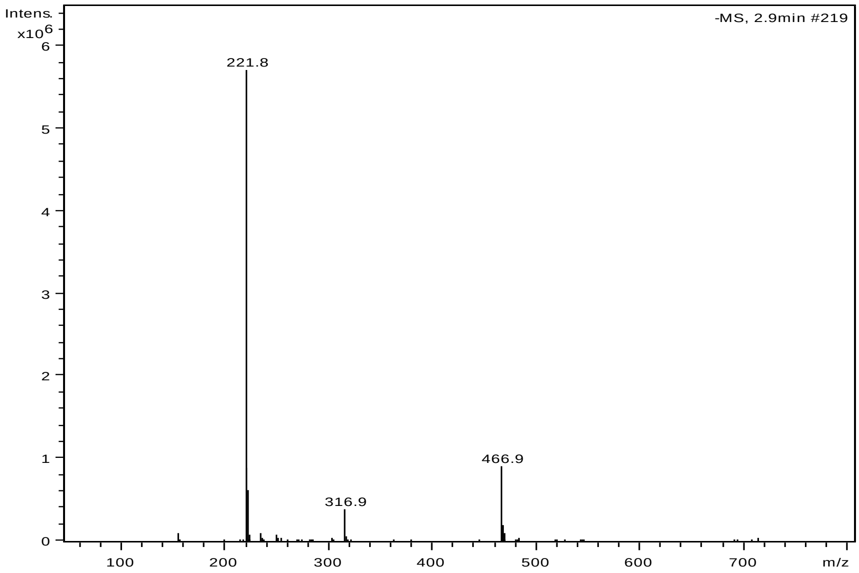
Intens.
x10^6
-MS, 2.9min #219
221.8
466.9
316.9
m/z

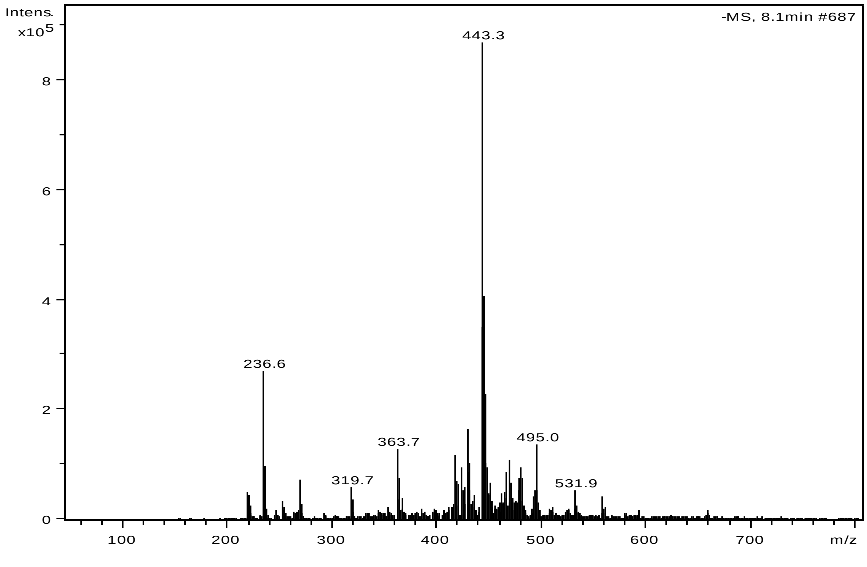
Intens.
x10^5
-MS, 8.1min #687
443.3
236.6
363.7
495.0
319.7
531.9
m/z

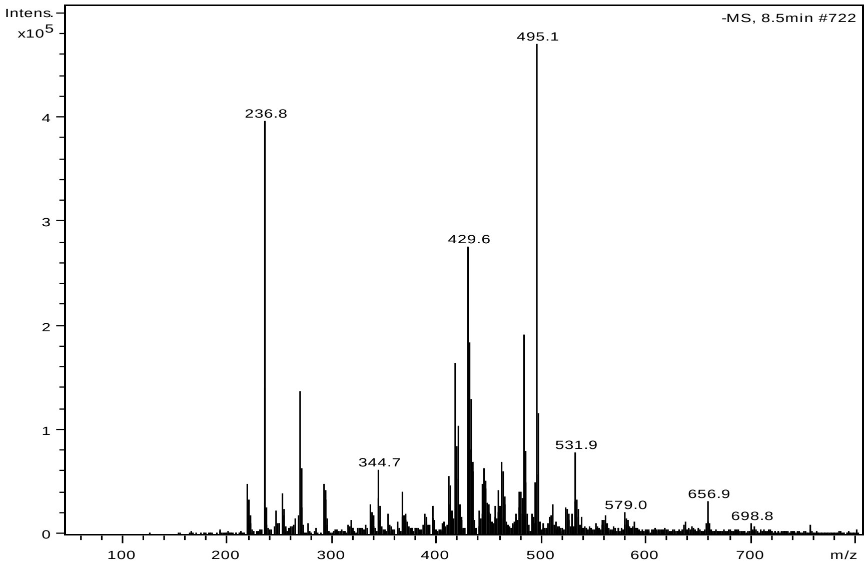

Intens.
x10^5
-MS, 8.5min #722
495.1
236.8
429.6
344.7
531.9
579.0
656.9
698.8
0
1
2
3
4
100
200
300
400
500
600
700
m/z

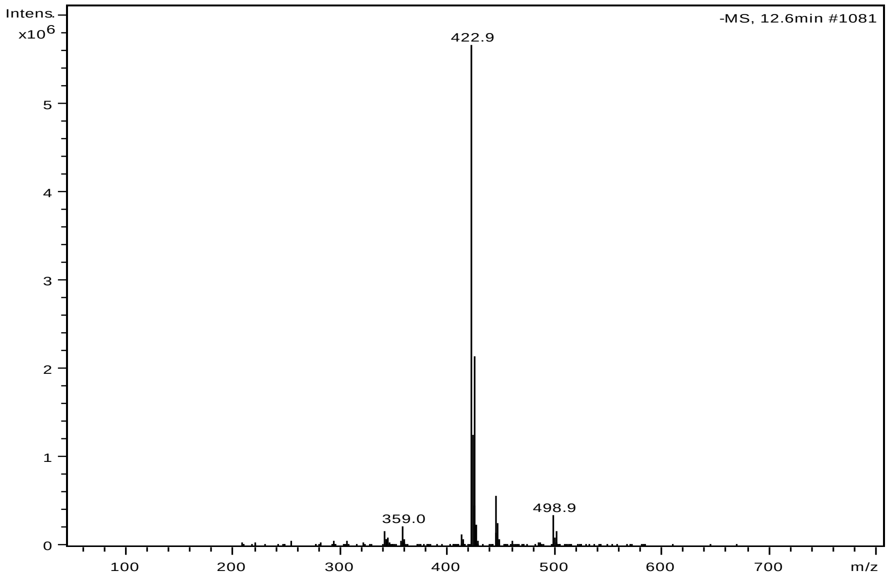

Intens.
x10^6
-MS, 12.6min #1081
422.9
359.0
498.9
0
1
2
3
4
5
100
200
300
400
500
600
700
m/z

Figure S10. a) LC chromatogram of the LC-MS analysis of the reaction mixture obtained after the reductive degradation of ion pair $(Octyl_3MeN)_2$.MB9 using $NaBH_4/NiSO_4$ in combination with Al-Ni alloy.

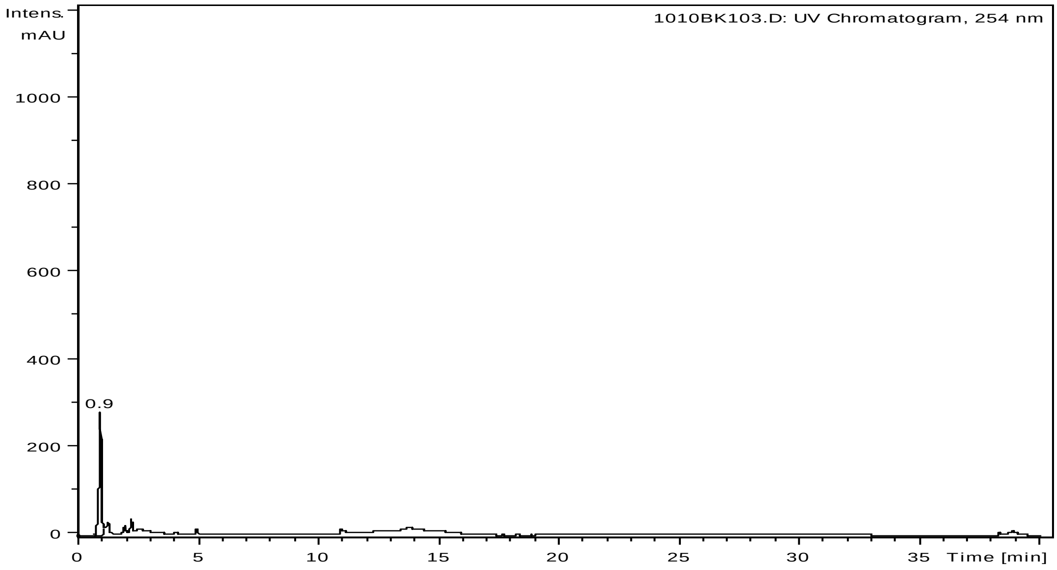

b) Qualitative LC-MS analysis of the reaction mixture obtained after the reductive degradation of ion pair $(Octyl_3MeN)_2$.MB9 using $NaBH_4/NiSO_4$ in combination with Al-Ni alloy.

RT (min)	*MW*	*Structure*
0.9	-	detected non-chlorinated structure

Note: Structures of the detected isomer compounds could not be to confirmed due to the lack of corresponding standard compounds.

c) Mass spectrum (EI, 70 eV) of analyzed component.

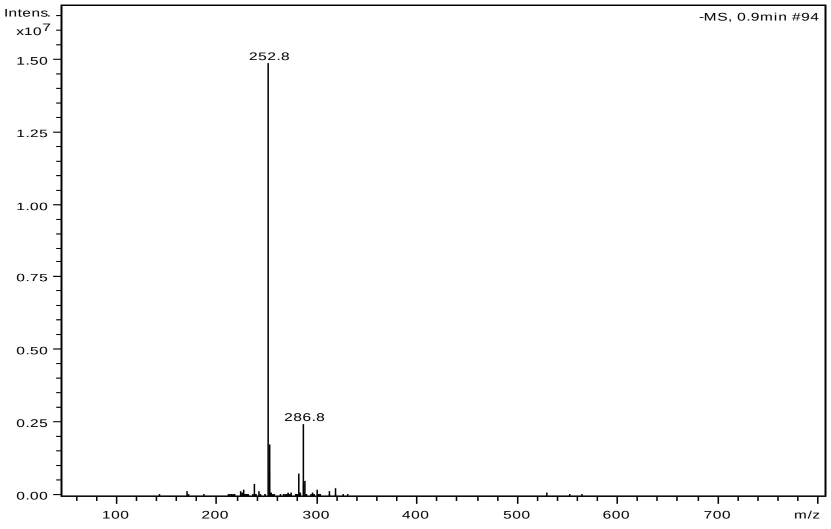

Figure S11. a) LC chromatogram of the LC-MS analysis of the reaction mixture obtained after the reductive degradation of ion pair (Luv. Mono LS)2.AY17 using $NaBH_4/NiSO_4$ in combination with Al-Ni alloy.

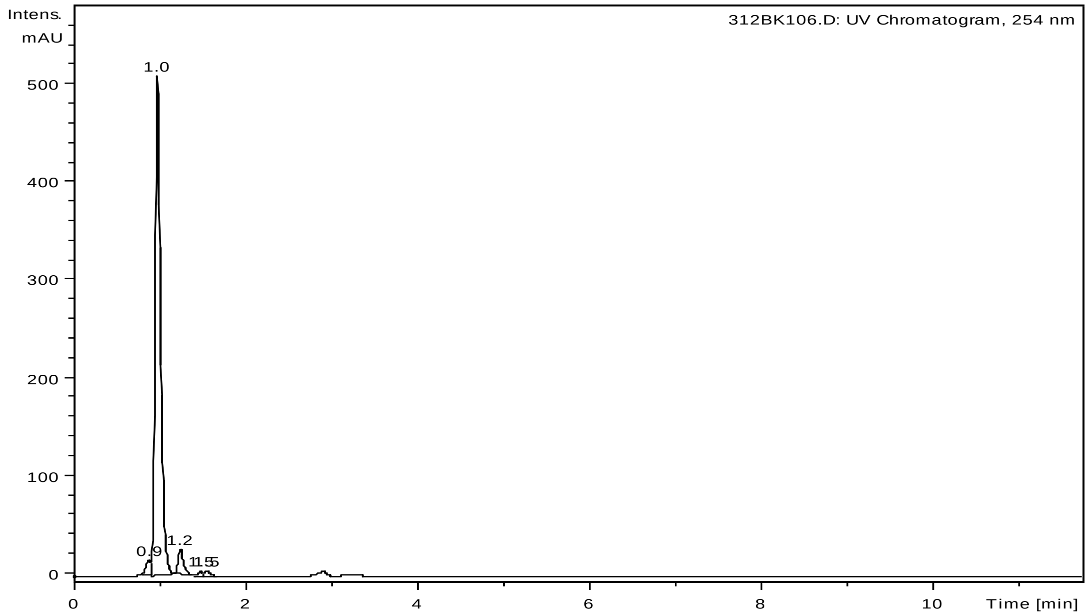

b) Qualitative LC-MS analysis of the reaction mixture obtained after the reductive degradation of ion pair (Luv. Mono LS)2.AY17 using $NaBH_4/NiSO_4$ in combination with Al-Ni alloy.

RT (min)	***MW***	***Structure***
1.0	173	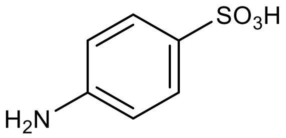

Note: Structures of the detected isomer compounds could not be to confirmed due to the lack of corresponding standard compounds.

c) Mass spectrum (EI, 70 eV) of analyzed component.

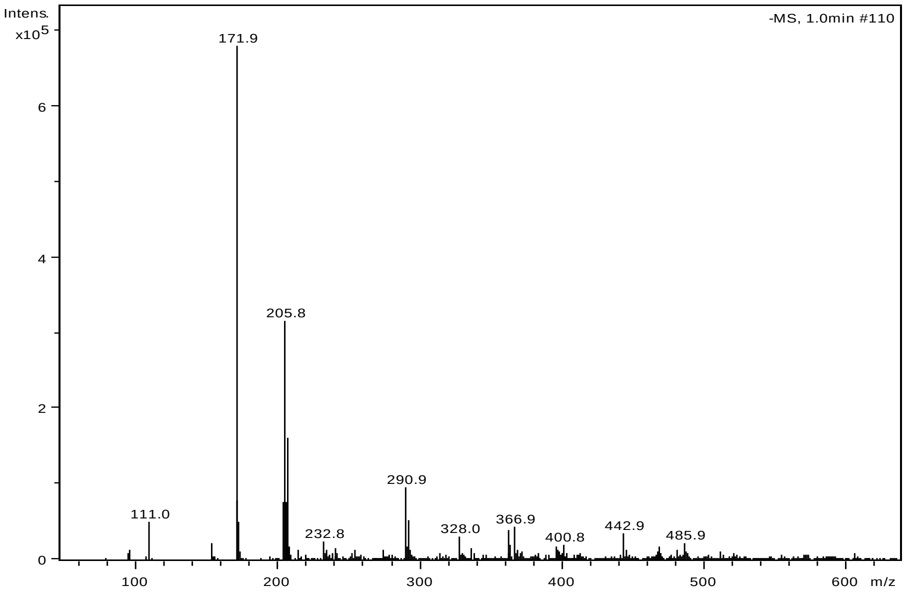

Figure S12. a) LC chromatogram of the LC-MS analysis of the reaction mixture obtained after the reductive degradation of ion pair $(Octyl_3MeN)_2$.AY17 using $NaBH_4/NiSO_4$.

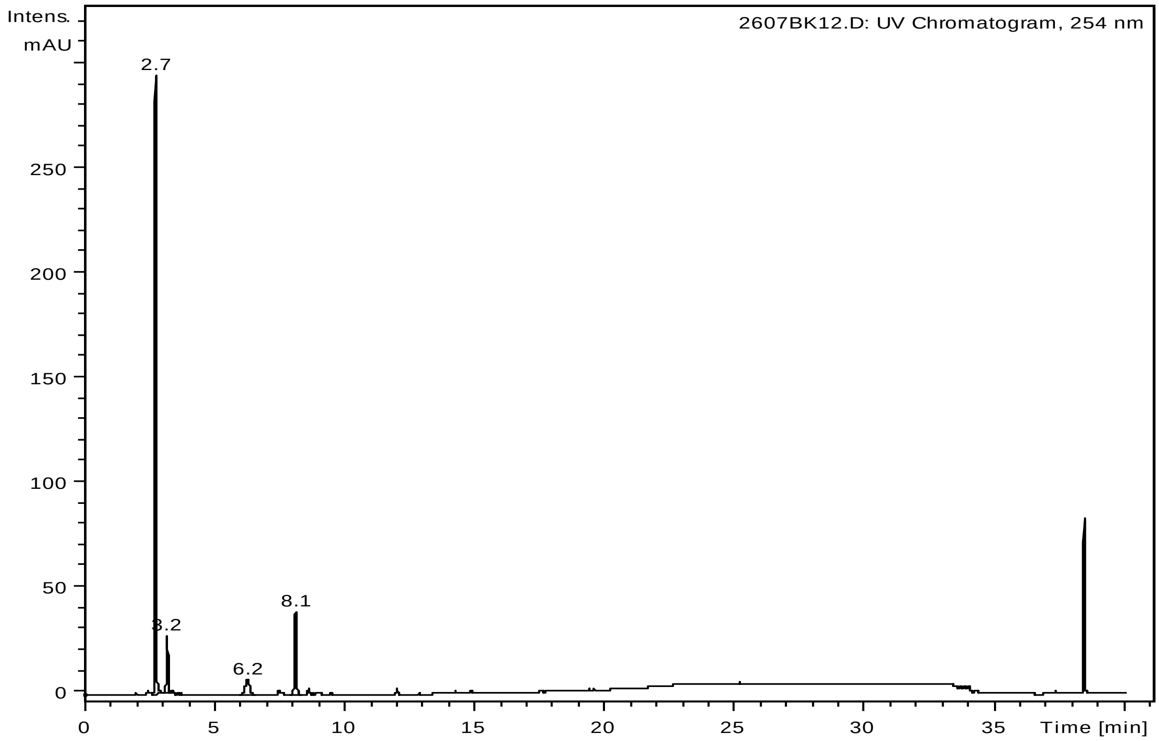

b) Qualitative LC-MS analysis of the reaction mixture obtained after the reductive degradation of ion pair $(Octyl_3MeN)_2$.AY17 using $NaBH_4/NiSO_4$.

RT (min)	*MW*	*Structure*
2.7	173	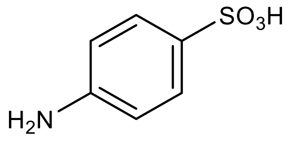
3.2	207	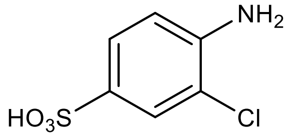
6.2	207	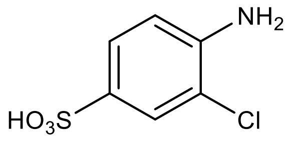
8.1	241	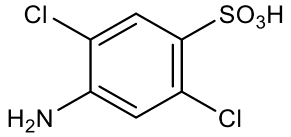

Note: Structures of the detected isomer compounds could not be to confirmed due to the lack of corresponding standard compounds.

c) Mass spectra (EI, 70 eV) of analyzed components.

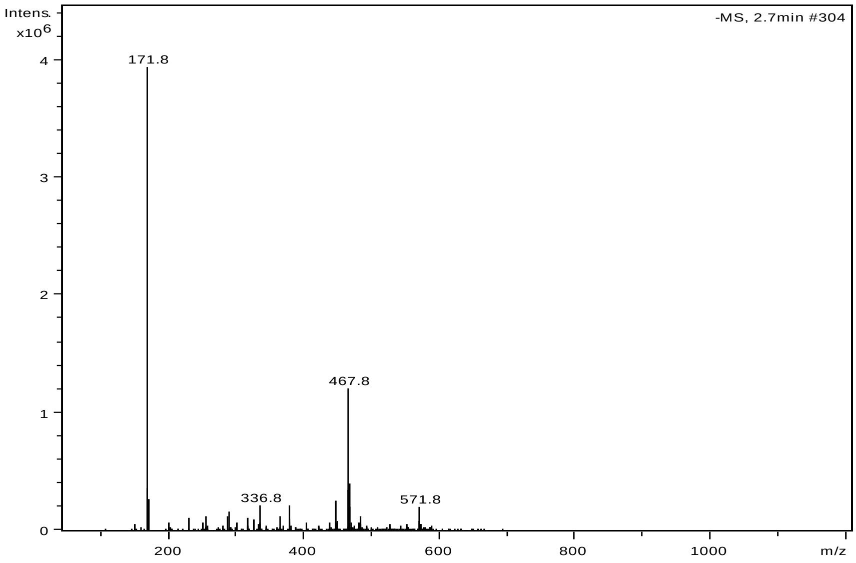

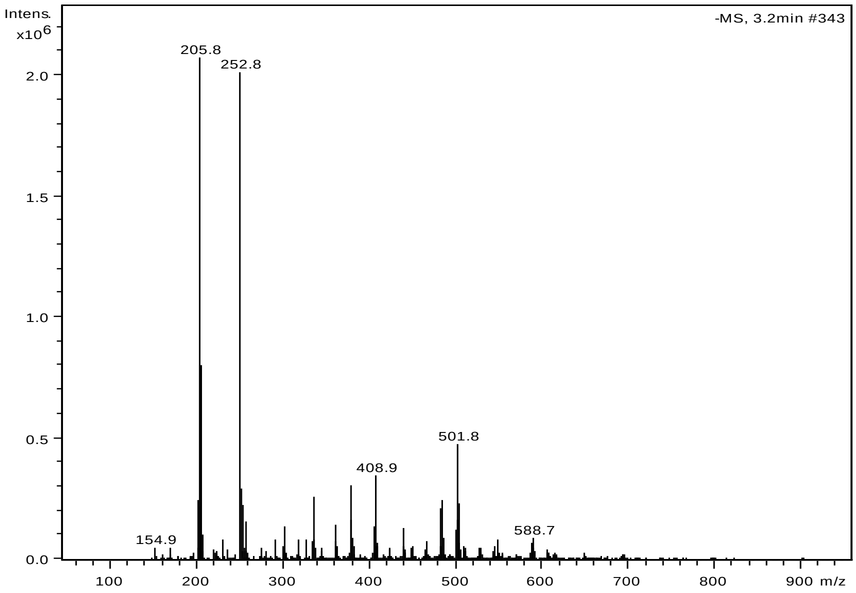

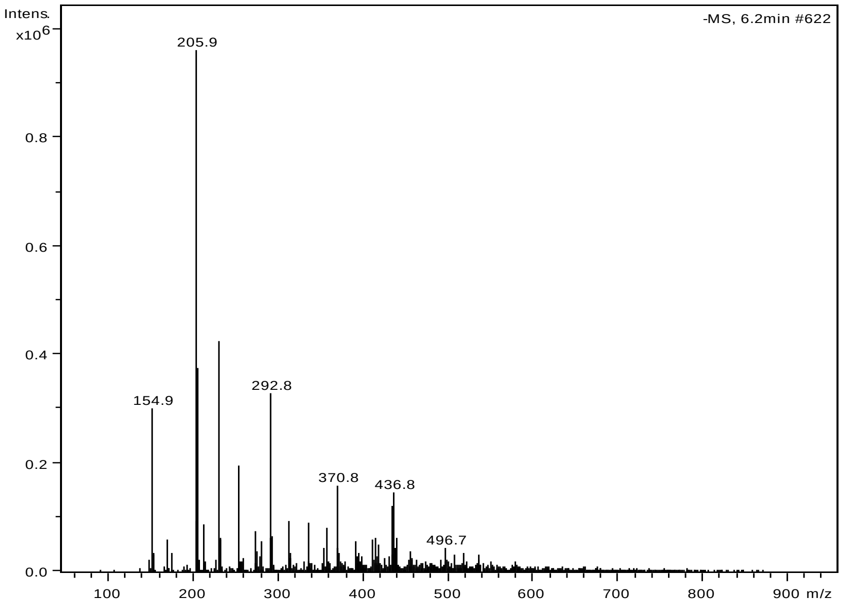

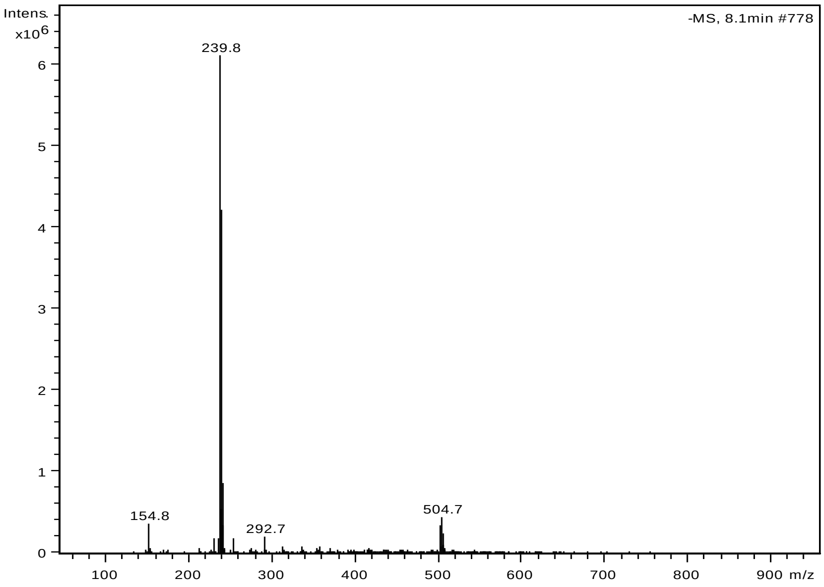

Intens.
x10^6
-MS, 8.1min #778
239.8
154.8
292.7
504.7
0
1
2
3
4
5
6
100
200
300
400
500
600
700
800
900 m/z

Figure S13. a) LC chromatogram of the LC-MS analysis of the reaction mixture obtained after the reductive degradation of ion pair (Luv.Mono LS)2.AY17 using $NaBH_4/NiSO_4$.

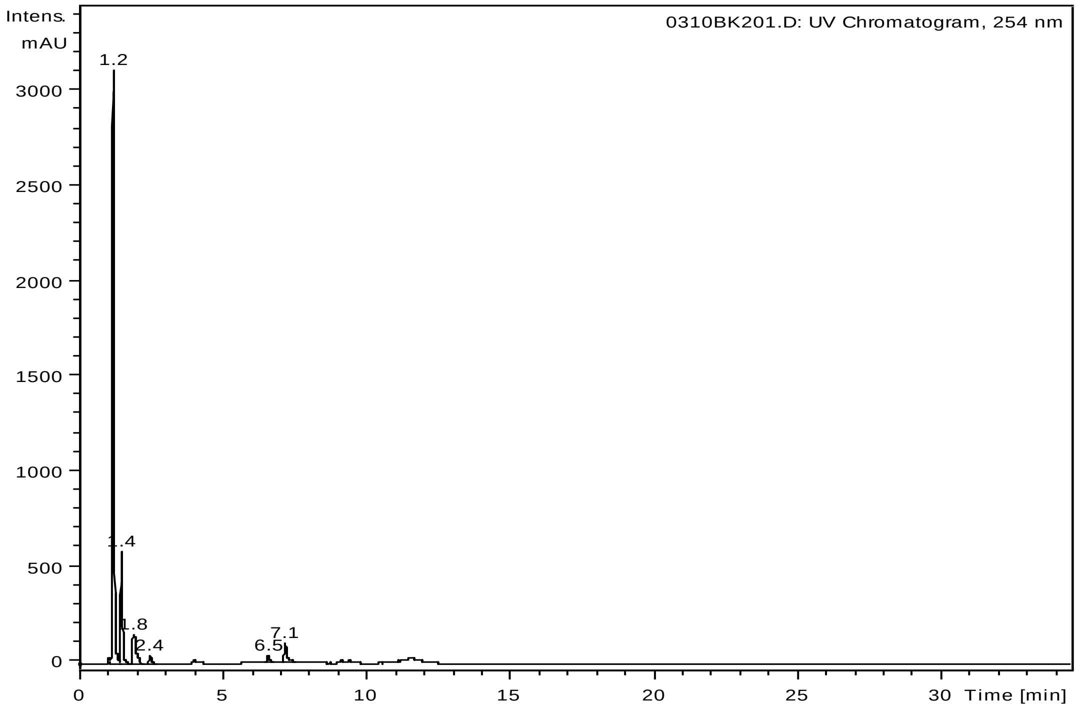

b) Qualitative LC-MS analysis of the reaction mixture obtained after the reductive degradation of ion pair (Luv.Mono LS)2.AY17 using $NaBH_4/NiSO_4$.

RT (min)	*MW*	*Structure*
1.2	173	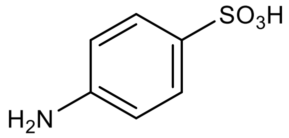
1.4	-	detected non-chlorinated structure
1.8	-	detected chlorinated structure
2.4	326	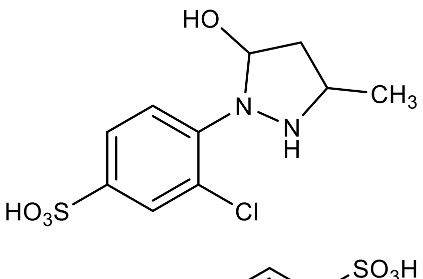
6.5	286	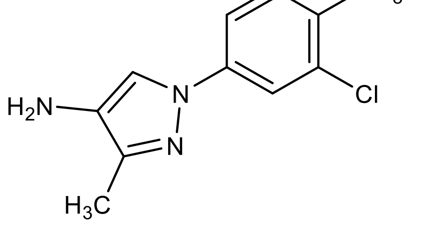

7.1	321	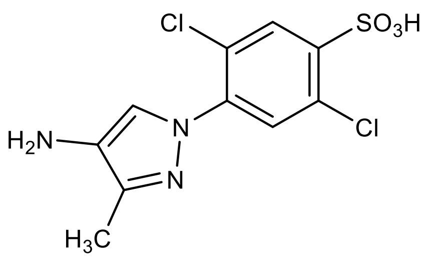

Note: Structures of the detected isomer compounds could not be to confirmed due to the lack of corresponding standard compounds.

c) Mass spectra (EI, 70 eV) of analyzed components.

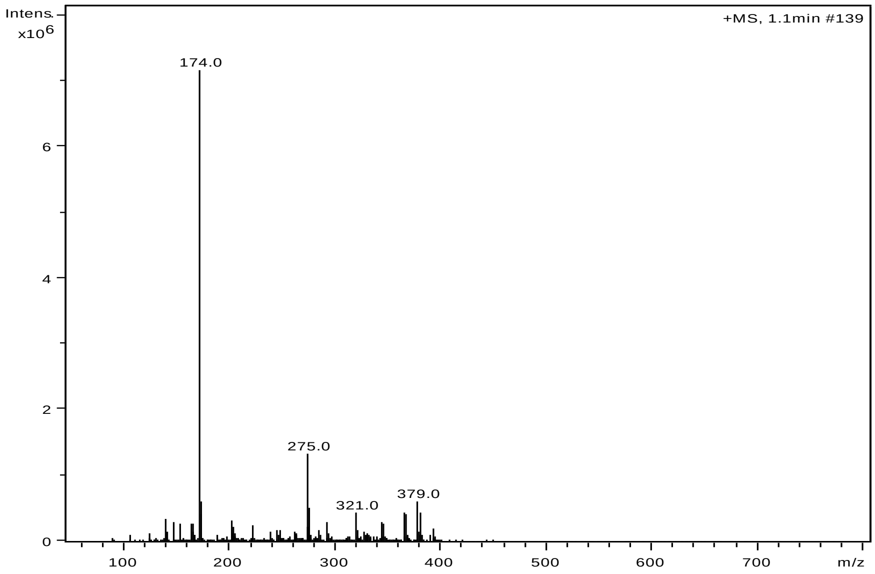

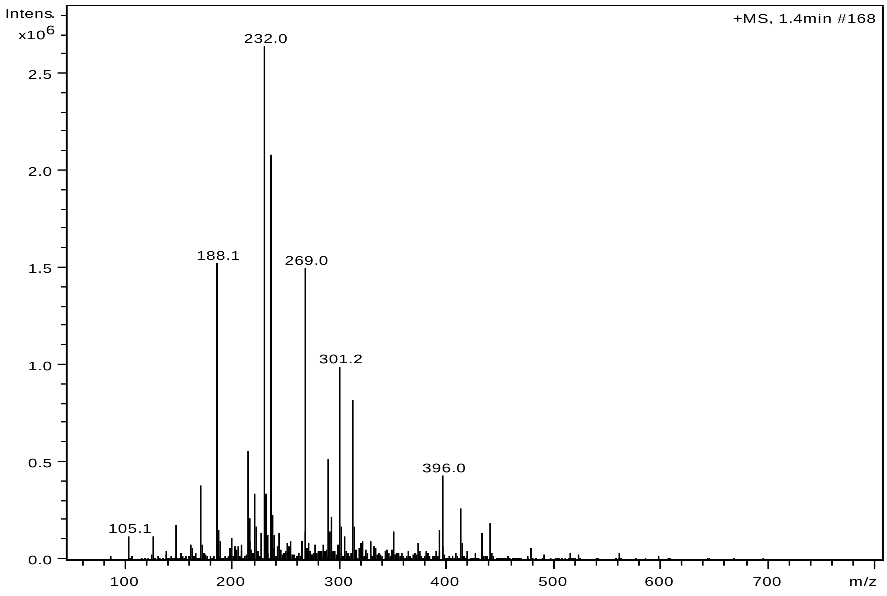

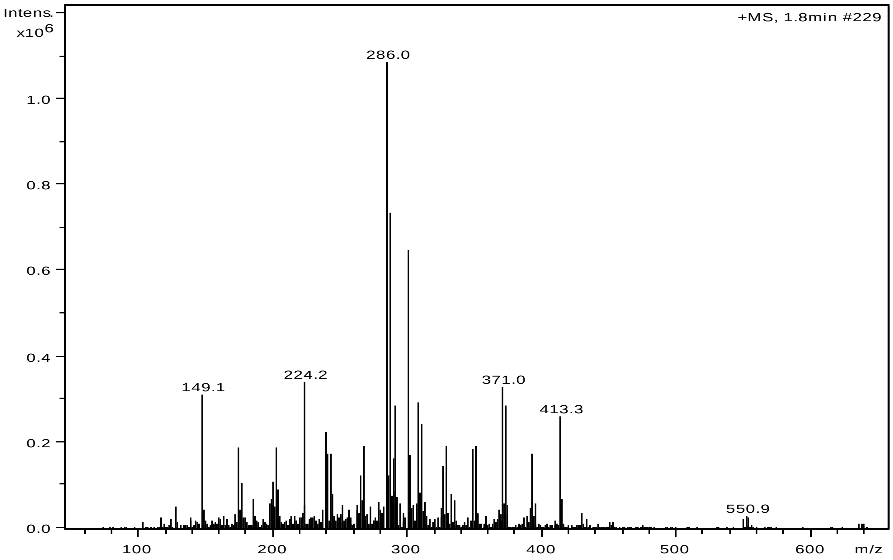
Intens.
x10^6
+MS, 1.8min #229
286.0
149.1
224.2
371.0
413.3
550.9
1.0
0.8
0.6
0.4
0.2
0.0
100
200
300
400
500
600
m/z

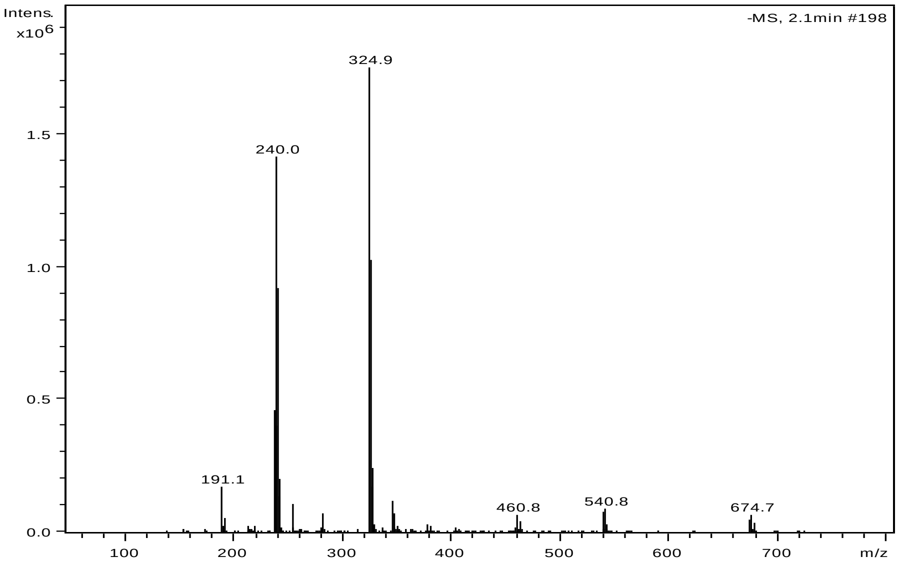
Intens.
x10^6
-MS, 2.1min #198
324.9
240.0
191.1
460.8
540.8
674.7
1.5
1.0
0.5
0.0
100
200
300
400
500
600
700
m/z

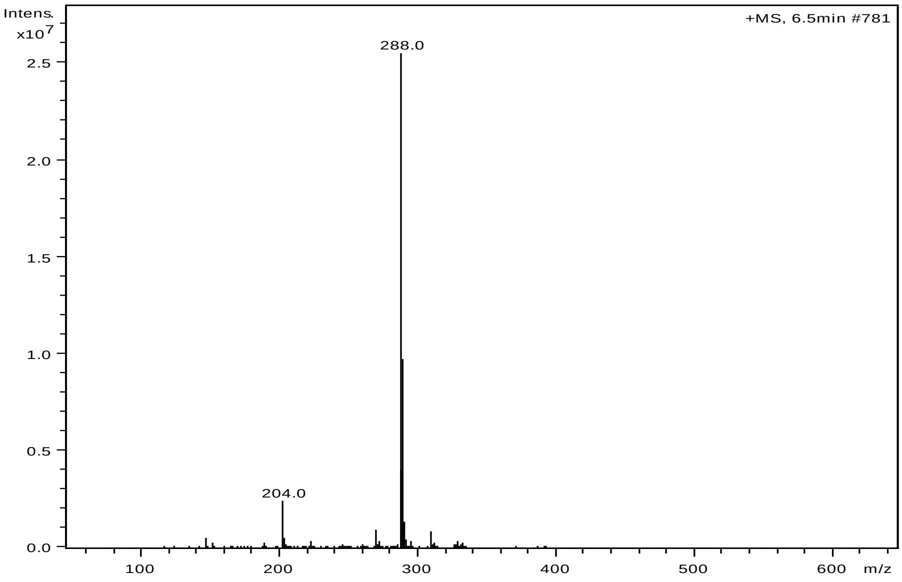
Intens.
x10^7
+MS, 6.5min #781
288.0
204.0
2.5
2.0
1.5
1.0
0.5
0.0
100
200
300
400
500
600
m/z

Figure S14. a) LC chromatogram of the LC-MS analysis of the reaction mixture obtained after the reductive degradation of ion pair $(Octyl_3MeN)_2$.MB9 using $NaBH_4/NiSO_4$.

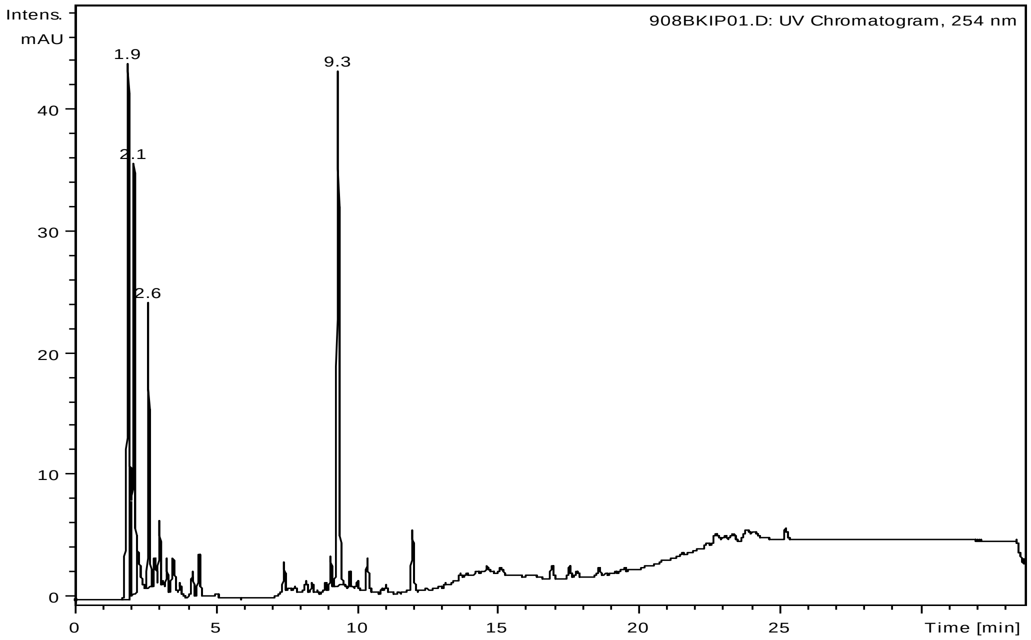

b) Qualitative LC-MS analysis of the reaction mixture obtained after the reductive degradation of ion pair $(Octyl_3MeN)_2$.MB9 using $NaBH_4/NiSO_4$.

RT (min)	*MW **	*Structure **
1.9	n.d.	n.d.
2.1	n.d.	n.d.
2.6	n.d.	n.d.
9.3	223	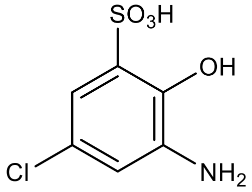

** n.d. – not determined*

Note: Structures of the detected isomer compounds could not be to confirmed due to the lack of corresponding standard compounds.

c) Mass spectra (EI, 70 eV) of analyzed components.

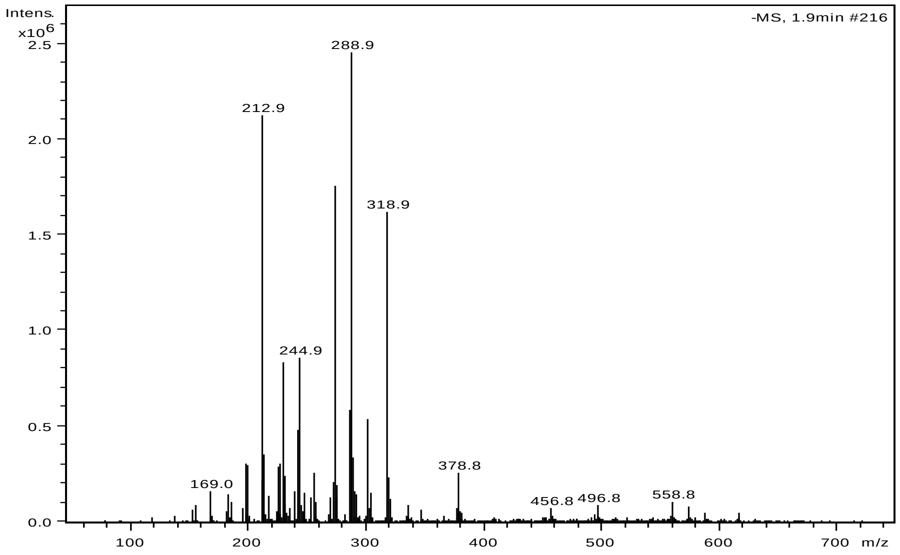

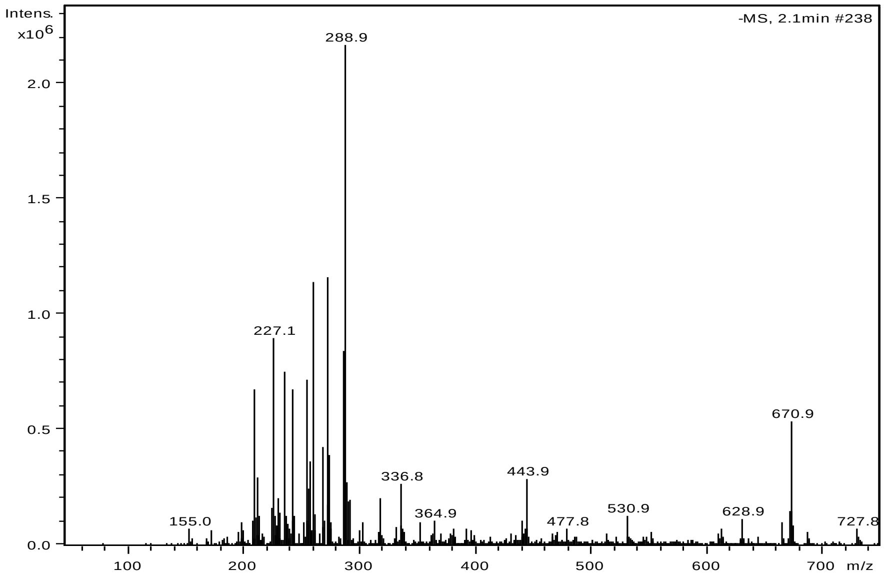

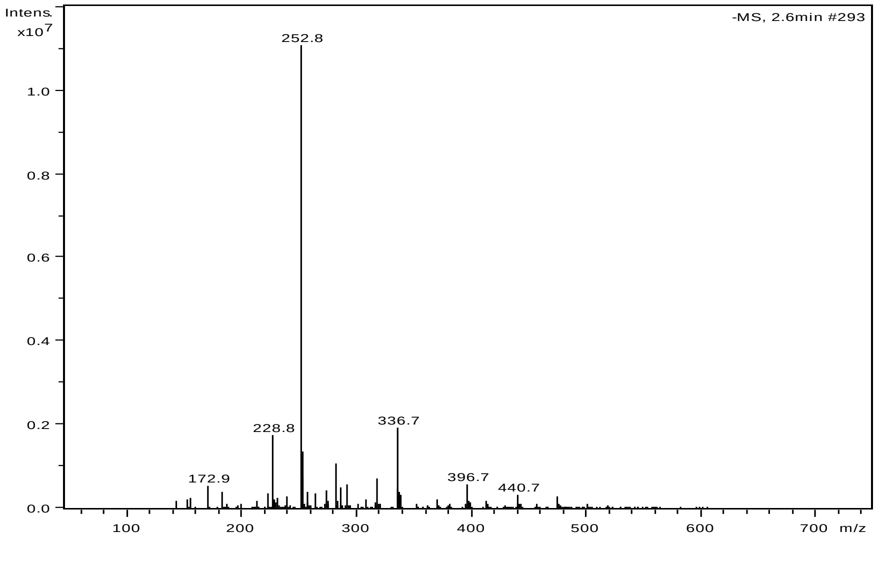

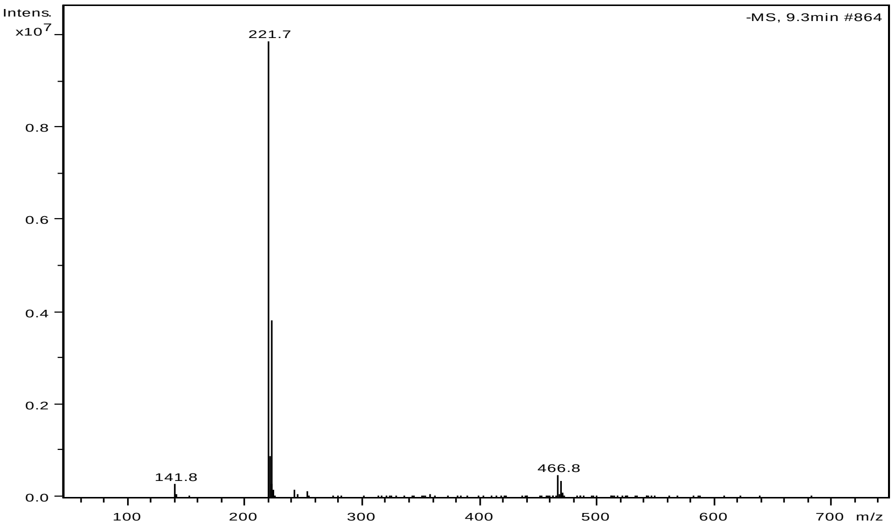
Intens.
x10^7
-MS, 9.3min #864
221.7
141.8
466.8
0.8
0.6
0.4
0.2
0.0
100
200
300
400
500
600
700
m/z
